# Clinicians’ experience of providing care: a rapid review

**DOI:** 10.1186/s12913-020-05812-3

**Published:** 2020-10-15

**Authors:** Maha Pervaz Iqbal, Elizabeth Manias, Laurel Mimmo, Stephen Mears, Briony Jack, Liz Hay, Reema Harrison

**Affiliations:** 1 School of Public Health and Community Medicine, UNSW Medicine, UNSW Sydney, Sydney, 2052 Australia; 2 School of Nursing and Midwifery, Centre for Quality and Patient Safety Research, Institute for Health Transformation, Deakin University, Geelong, Australia; 3 Sydney Children’s Hospitals, Network, Sydney, NSW Australia; 4 Hunter New England Medical Library, New Lambton, NSW 2350 Australia; 5 Strategic Reform and Planning Branch, NSW Ministry of Health, St Leonards, NSW 2065 Australia; 6 Economics and Analysis, Strategic Reform and Planning Branch, NSW Ministry of Health, St Leonards, NSW 2065 Australia

**Keywords:** Value based Health care, Clinician experience, Rapid evidence assessment

## Abstract

**Background:**

Health care services internationally are refocussing care delivery towards patient centred, integrated care that utilises effective, efficient and innovative models of care to optimise patient outcomes and system sustainability. Whilst significant efforts have been made to examine and enhance patient experience, to date little has progressed in relation to provider experience. This review aims to explore this knowledge gap by capturing evidence of clinician experience, and how this experience is defined and measured in the context of health system change and innovation.

**Methods:**

A rapid review of published and grey literature review was conducted utilising a rapid evidence assessment methodology. Seventy-nine studies retrieved from the literature were included in the review. Fourteen articles were identified from the grey literature search and one article obtained via hand searching. In total, 94 articles were included in the review. This study was commissioned by and co-designed with the New South Wales, Ministry of Health.

**Results:**

Clinician experience of delivering health care is inconsistently defined in the literature, with identified articles lacking clarity regarding distinctions between experience, engagement and work-related outcomes such as job satisfaction. Clinician experience was commonly explored using qualitative research that focused on experiences of discrete health care activities or events in which a change was occurring. Such research enabled exploration of complex experiences. In these contexts, clinician experience was captured in terms of self-reported information that clinicians provided about the health care activity or event, their perceptions of its value, the lived impacts they experienced, and the specific behaviours they displayed in relation to the activity or event. Moreover, clinician’s experience has been identified to have a paucity of measurement tools.

**Conclusion:**

Literature to date has not examined clinician experience in a holistic sense. In order to achieve the goals identified in relation to value-based care, further work is needed to conceptualise clinician experience and understand the nature of measurement tools required to assess this. In health system application, a broader ‘clinician pulse’ style assessment may be valuable to understand the experience of clinical work on a continuum rather than in the context of episodes of change/care.

## Background

Healthcare services internationally are refocussing care delivery towards patient centred, integrated care that utilises effective, efficient and innovative models of care to optimise patient outcomes and system sustainability [1]. Adapting to change is therefore intrinsic to healthcare delivery to meet the emerging systems priorities, and to respond to changing population demographics including longer life expectancy, enhance health system sustainability, enable the management of increasingly complex and chronic health conditions, and optimise technological and service delivery innovations [2, 3].

Value-based healthcare (VBHC) programs across health systems internationally include goals of ensuring positive patient and clinician experiences [4, 5]. Whilst optimal measures of patient experience are widely debated, the inclusion of measures such as Patient Reported Outcomes and Experience Measures (or Patient Reported Outcomes Measures and Patient Reported Experience Measures), into routine health system data collection processes is now well established. Yet there remains an evidence gap regarding how to measure and understand the clinicians’ experience comprehensively. Experience is a multi-dimensional concept and many factors can contribute to this concept. Experience can be the knowledge and understanding which is gained after an event, which may contribute to the formation of attitudes, beliefs and perceptions, or may be the process of living through or undergoing an event. There is also an affective or emotional component to the concept of experience [6].

Unlike patient experience, measurement of clinician experience of providing care generally occurs in relation to a specific change project, with particular focus given to clinician engagement, for example in promoting the use of evidence in clinical practice [7]. A further body of research has provided evidence of psychological experiences associated with clinical work such as burnout, depression, stress, fatigue and experiences of stressful events such as clinical error [8–10]. These data provide important information about the impacts of clinical work but are not comparable to the information sought from patients [11] holistically about their experiences.

In the New South Wales (NSW) Health system in Australia, value-based healthcare means continually striving to deliver care that improves the health outcomes that matter to patients; experiences of receiving care; experiences of providing care; and the effectiveness and efficiency of care [12], reflecting the Quadruple Aim framework [4, 13]. The NSW Health approach recognises the critical role of the health care workforce in providing enhanced health care. Towards the VBHC agenda, the present review commissioned by NSW Health, aimed to explore this knowledge gap by reviewing evidence of clinician experience, and how this experience is defined and measured in the context of health system change and innovation, by addressing the following research questions:

1. How has clinician experience of delivering health care been defined in the published and grey literature?
2. What survey instruments and measures of clinician experience have been developed, and applied to evaluate the impact of a health system change or monitor health system performance?
3. How have clinician experience measures been used to assess the impact of health system change on the experience of providing care?

## Methods

A rapid evidence assessment (REA) methodology was utilised to explore the literature. A REA is a research methodology which uses the same methods and principles as a systematic review but makes concessions to the breadth or depth of the process, in order to suit a shorter timeframe and address key issues in relation to the topic under investigation [14–16]. In this instance, REA was selected as an appropriate method to explore the breadth of the topic and disparate nature of work in clinician experience of providing care, with the objective to provide a balanced assessment of what is already known about this issue. In addition, the Preferred Reporting Items for Systematic Reviews and Meta-Analyses—PRISMA statement—was used to guide the reporting of this rapid review [16].

### Eligibility criteria

The articles were included if they met the following inclusion criteria:

- *Types of publication:* Publications were eligible that are available in English and reported original primary empirical or theoretical work published in the last 10 years (January 2009–June 2019).
- *Types of settings:* Any healthcare setting, including but not limited to public or private hospitals, day procedure centres, general practice or other primary/community care in countries including Australia, England, New Zealand, Northern Europe and North America. These countries were selected on the basis of the similarities of their health care environments and systems.
- *Types of study design:* Conceptual, theoretical, quantitative or qualitative studies of any research design.
- *Interventions:* Studies examining changing/new/innovative healthcare delivery that includes but is not limited to the introduction of technologies of other innovations; introduction of new models of care or redesign of care processes; workflow and service restructure or the introduction of new programs or policies.

#### Outcomes

Experience data from clinicians defined as self-reported information about what happened to any given clinician whilst providing health care.

### Literature search

#### Study identification

A range of text words, synonyms and subject headings were developed for the three major concepts in this review of *clinician experience, change* and health care (Key search terms: chang*, transform*, clinician engagement, implement*, introduce*, clinician experience, ways of working, disrupt*, models of care) (See search strategy- Additional file 3)*.* These text words, synonyms and subject headings were used to undertake a systematic search of two electronic databases that index journals of particular relevance to the review topic (Medline and PubMed). Reference lists of the included full text articles were also hand-searched to capture relevant published material. The grey literature (e.g. reports and papers published by government departments, intragovernmental agencies, public or private health service providers, non-government agencies, professional bodies, advocacy groups etc.) were identified by searching the websites of relevant organisations. Literature identified was assessed along with the papers from the database searches.

#### Study selection and data extraction

Articles were managed using a reference-management software (Covidence) and duplicates were removed. Two reviewers (EM; LM) independently screened the titles and abstracts or the executive summaries for grey literature. Title and abstract screening review that fulfilled the inclusion criteria, were selected to undergo a full-text review. Inclusion criteria were then independently applied to the full text articles by two further reviewers (RH; MPI). A team member (EM) then conducted a face validity check of the identified material. Disagreements were resolved through final discussion between the whole of the review team.

#### Narrative data synthesis

Findings were analysed using a narrative empirical synthesis, based on the research questions. The narrative approach [17] was used to synthesise the qualitative and quantitative findings. Initial descriptions of eligible studies and results were tabulated (Additional files 1 and 2).

## Results

### Results of the search

**Fig. 1:**
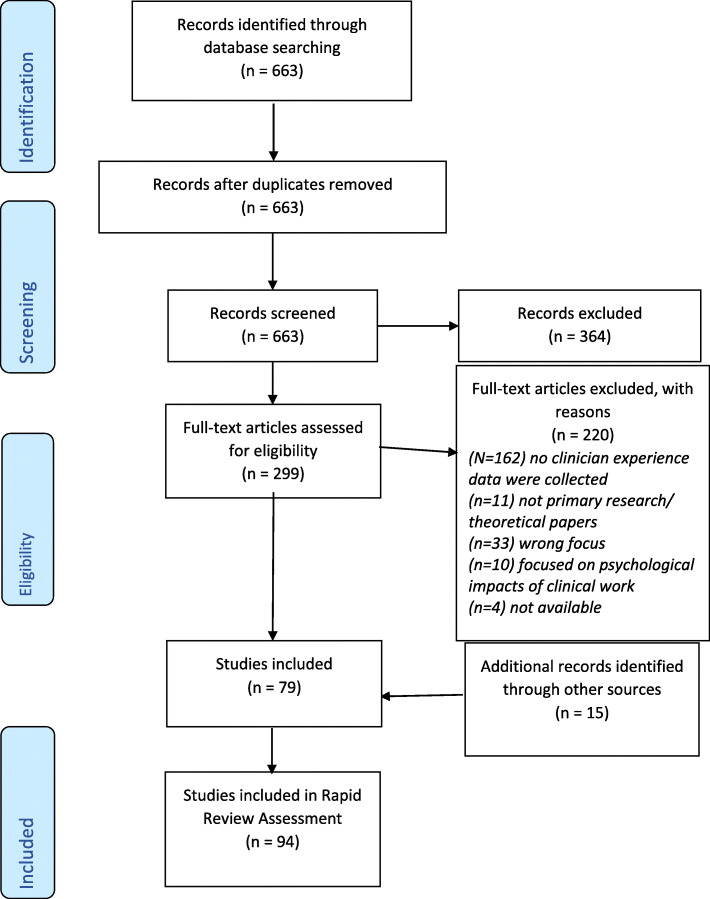
PRISMA Flow Diagram for Clinicians’ Experience of Providing Care: A Rapid Review

After removing duplicates, 663 records were identified. Title and abstract screening review resulted in 299 publications that fulfilled the inclusion criteria. Seventy-nine studies were included in the review. Fourteen articles were identified from the grey literature search and 1 article obtained via hand searching. In total, 94 articles were included in the review (see Fig. 1).

### Excluded studies

Studies were excluded at the full-text review stage because they did not meet the inclusion criteria in that no clinician experience data were collected (*n* = 162), not being primary research or theoretical papers (*n* = 11) or had a wrong focus (*n* = 33) or focused on only psychological impacts of clinical work (*n* = 10) or were not available (*n* = 4).

### Review findings

Articles emerged from Articles emerged from Australia (*n* = 21), UK (*n* = 16), US (*n* = 13), Canada(*n* = 10), Denmark (*n* = 8), Sweden (*n* = 6), Spain (*n* = 2), Switzerland (*n* = 2), Italy (*n* = 2), Norway (*n* = 3), France (*n* = 2), New Zealand (*n* = 2), The Netherlands (*n* = 2), Singapore (*n* = 1), Hong Kong (*n* = 1), Northern Ireland (*n* = 1), Israel (*n* = 1) and Germany (*n* = 1). Articles reported data from the health system level (*n* = 8), across multiple areas within one or more hospitals (*n* = 60), specific specialities within hospitals (*n* = 10), integrated care settings (*n* = 8), primary care (*n* = 6), community health care (*n* = 1), and ambulance (1). Diverse health care settings were utilised, comprising mainly inpatient hospital settings which included intensive care, perioperative care, emergency care and maternity settings. Articles were qualitative (*n* = 41), quantitative (*n* = 24) or mixed- or multi-methods (*n* = 31). Health professional groups examined in the studies mainly involved nurses and doctors, but a number of studies also included allied health professionals or pharmacists. Other health professional discipline groups examined involved midwives, and dietitians.

Past research on clinician experience comprised qualitative research designs involving the conduct of interviews and focus groups. Few studies involved the conduct of observational work (*n* = 6), which would have enabled examination of clinical experiences in actual practice. Similarly, there were few studies that involved the conduct of survey studies (*n* = 24) which often examined the psychometric properties of variables underpinning clinician experiences.

#### Question 1: how has clinician experience of delivering health care been defined in the literature?

Clinician experience has largely been understood as self-reported information presented by clinicians about how they practise patient care, the activities they undertake and how providing care makes them feel. In some instances, observational data were also collected and synthesised to capture the events that occurred within care provision [18–22]. Two dominant approaches to conceptualising clinician experience emerged, which included subjective reports of past experiences and of the process of undertaking a change that were evident from the question scope and content of the included studies. Questions commonly explored clinicians’ perception of the need or justification for change, their perceived impact of change on personal care practices, and their impressions of contributing to change. For example, this impact was considered when examining clinician experiences to meet a four-hour target in the Emergency Department through a new model of care [23]. Similarly, when redesigning post-natal care, midwives were asked to discuss their experiences of the changes made [24].

The literature indicated there was overlap between, 1) clinician experience of delivering care, 2) health workforce job satisfaction and 3) clinician engagement. It was apparent that self-reported experiences of providing care were closely linked to both resulting job engagement and satisfaction [19, 25–29]. The nature of this relationship was not sufficiently examined to draw conclusive findings regarding the relationships between these variables. For example, lack of clinician engagement was identified in the negotiation of professional boundaries among clinicians’ during health services change [30]. This lack of engagement reproduced inequalities among professional groups and prevented some groups from participation in service change. However, there was little information about the relationship between engagement and experience, such as if clinicians’ experiences had led to a lack of engagement or whether lack of engagement further affected clinician experience [30] Interviews with doctors in cancer care services demonstrated their organization comprised a work system, which consisted of a set of specific actions and narrow-focused tasks. This experience underestimated the emotional components of patient-doctor encounters, which impacted job satisfaction. The creation and maintenance of genuine patient-doctor relationships were therefore more difficult to attain, leading to perceptions of failed doctor encounters with patients, on behalf of the doctors [31].

A number of indicators emerged from the literature as contributing to positive or negative clinician experiences. Indicators of positive clinician experiences were linked to clinicians’ attributes, the environment in which they worked and system changes. Active patient participation in health care contributed to positive clinician experiences, while clinicians’ respect for each other’s competencies and valuable contributions to patient care influenced their ability to collaborate effectively [26, 32]. The development of trust among team members of different disciplines was regarded as essential for effective clinician experience [33]. The leadership style of clinicians was a positive indicator of experiences, especially when promoting interdisciplinary practice [33, 34]. Senior management support for how clinicians conducted their work [35], the availability of ongoing education and training tailored to the needs of various professional groups [36], and the presence of an organisational culture that addressed patient care needs facilitated positive clinician experiences [37]. In addition to this support, clinicians who practised patient-focused models of care expressed enhanced clinicians’ experiences. For example: pharmacists who were in patient-focused practice settings were more likely to seek opportunities to collaborate with physicians to discuss prescribing practices [38].

Indicators of negative clinician experiences included tensions when balancing professional responsibilities and quality of patient care. Clinicians reported that complex arrangements in which professional responsibilities were unclear negatively impacted the quality of the care they could provide and their experience of providing care [27]. In addition to this, there was a discussion on the impact of a fragmented system of care in which interprofessional members constituted a team or that multiple teams were responsible for patient care. This fragmented care system often created confusion regarding patient responsibility and clinical decision making, which had a negative impact on the clinicians’ experience [39, 40]. The hierarchical structure that was particularly apparent in hospital settings was reported to adversely affect clinician experience; such that communication practices were ineffective in terms of disseminating information about project awareness and knowledge transfer which as a result impacted clinician engagement [35].

#### Question 2: what survey instruments and measures of clinician experience have been developed, and applied to evaluate the impact of a health system change or monitor health system performance?

The under-developed conceptualisation of clinician experience was reflected in the lack of survey research in this field. Twenty studies (18 from the database search and two from the grey literature) included survey methods to capture clinician experience data, with no single survey instrument widely adopted within these studies [28, 29, 41–57]. Of the identified studies, only two survey studies sought to assess clinician experience of providing care beyond a specific change event and using a survey instrument explicitly capturing experience outcomes [42, 57]. Surveys were cross-sectional or over short time, with no longitudinal research emerging.

Only one study sought to develop and validate a clinician experience measure [57]. This survey was also utilised in the grey literature [58]. Experience of work was defined as encompassing a number of facets which are included in the Picker Staff/Employee Questionnaire [59]. The items within the Picker surveys indicate that work experience data comprise a range of self-reported information about the work environment interactions within this experience, perceptions of environment and interactions, and satisfaction with the work environment. The United Kingdom (UK) National Health Service (NHS) has engaged Picker Europe to undertake a staff survey across the NHS each year, which comprises a core composite and additional optional elements for individual services or groups within the NHS to use such as leadership assessments [60].

Studies often reported experience data within another outcome including: clinician engagement, team climate, emotional exhaustion, information systems expectations or safety attitudes, with some degree of experiential data within each of these studies [28, 29, 47, 48, 50, 61]. Definitions of clinician engagement varied substantially, and its relationship with experience was poorly defined. Some studies therefore captured engagement using validated measures and then added on specific items to capture experiential data that were linked to engagement. For example, in a study by Dellve et al. [28], clinician engagement was conceptualised as *‘attitudes toward engagement in organizational development, work engagement as a cognitive state, and clinical engagement behavior in developing patient safety and quality of care in practice* [28]*.* Clinician engagement behaviour was captured on two scales; a patient safety scale (consisting of four items) and a quality of care scale (consisting of three items) [28]*.* The items in each scale ask clinicians about their experiences of engagement in safety and quality activities or programs, but are limited to key behaviours, which do not encapsulate the holistic clinician experience of providing care.

Broader use of the term ‘engagement’ was demonstrated in a study that discussed nurse engagement with an initiative utilising a survey [61]. In this survey study, outcome variables included data of clinicians perceptions of the change, buy-in, experiences of impacts on the caregiving process and satisfaction [61]. Press Ganey (2017) made a link between patient experience, workforce engagement and financial outcomes for health care organisations in their strategic paper on investigation of engagement, morale and working conditions of staff in Perth Children’s Hospital [49]. Their research utilised two survey tools that contributed to the Perth Children’s Hospital Investigation: physician engagement and the Practice Environment Scale–Nursing Work Index (PES–NWI) [49, 62]. The PES-NWI was derived from the Nursing Work Index (NWI) and developed specifically to capture the hospital practice environment. Items of the PES-NWI consist of five subscales that capture experiences in relation to key aspects of the work environment of hospitals: “*Participation in Hospital Affairs,” “Foundations for Quality of Care,” “Manager Ability, Leadership and Support of Nurses,” “Staffing and Resource Adequacy,” and “Collegial Nurse-Physician Relations”* [63]. The Press Ganey report focuses on subscale data in relation to safety and quality outcomes, reporting a relationship between a positive work environment, engagement and safety and quality of care [49].

In a group of nine studies, authors developed their own surveys in the absence of existing validated measures to capture experiential data from clinicians about current processes of care provision or specific changes to care provision, including the introduction of an electronic health record (EHR), the treatment of sepsis, and redesign of observational charts [24, 44–46, 51–54, 56]. In these studies, surveys were often lengthy, with multiple components. Two multi-instrument studies combined validated measures and purposively developed items [43]. In the first, the Karasek Job Content Questionnaire, the Nursing Work Index-Extended Organization (NWI-EO), the SF-12 Health Survey, 51 researcher-developed items were used to explore the impact of a departmental relocation on psychosocial job characteristics, perceived health, and psycho-organisational constraints amongst health care workers [43]. By including validated measures incorporating clinician engagement, impact of work on health and job satisfaction, this study provided a more thorough assessment of clinician experience. The study highlighted that in order to capture holistic experience data, it was important to synthesise a range of validated and purposively developed survey instruments. Similarly, a study of staff experiences of closing a psychiatric ward captured uncertainty and self-efficacy using validated tools, and experiences of perceived functioning on the ward through a researcher-developed tool [55].

#### Question 3: how have clinician experience measures been used to assess the impact of health system change on the experience of giving care?

Clinician experience was primarily captured for the purpose of providing feedback on a single change project or issue, to understand its perceived value, impacts on care delivery and factors that may impact its uptake and ability to achieve maximum benefits realisation. For example introduction of a new technology such as electronic medical record (EMR) across a health service [56] or to local-level quality improvement projects [54] was captured. Far fewer studies examined holistic clinician experience and focused on exploring experience of providing care across a range of events, interactions and points in the care-giving process [57]. The body of evidence strongly indicated that clinician experience was captured and valued appropriately for the purpose of improving care, but that consideration of the experience of providing care on clinician’s personal and professional lives was not a key driver of the data collection [64]. In qualitative work, clinicians were recruited as key informants about the use of a new system, technology or model of care [65, 66].

The grey literature provided several examples of evaluations of system level changes and the way that clinician experiences were measured. These evaluations were used to assess the impacts beyond a specific context such as a single ward, department, or service. Research designs that included a survey around benefits realisation in terms of clinical and process outcomes, coupled with qualitative interviews to capture experience data were apparent in many articles as a strategy to capture data from clinicians about change projects [67, 68]. Other reports included interviews with system-level stakeholders and clinicians were used, along with document and observational analysis, to understand the model of change applied, its value and the factors impacting implementation [69].

Five articles from the retrieved literature captured clinician experience more broadly, rather than in relation to a change project [31, 57, 60, 62, 70]. The focus of this work was primarily around the changing role of clinicians in contemporary health care. Several reports of investigations into poor quality care or stemming from such investigations were identified in the grey literature that discussed the issue of clinician experience of care provision and its intersection with safety, quality and patient experience [62].

## Discussion

Clinician experience is closely connected to work and psychological experiences. This review highlighted a lack of clarity regarding the relationship between clinician experience and the related constructs of engagement and job satisfaction that were commonly captured alongside experience data in the included studies. The review identified a large number of qualitative studies exploring clinician experiences in the context of changes occurring in health services and system, with fewer (*n* = 24) survey studies, and just two articles that reported a service or system-level clinician experience survey [57, 60]. In these two studies, the Picker Staff/Employee Questionnaire was adapted and administered.

The concept of clinician experience is not well-defined in the research literature. The predominant use of self-reported data collection methods and of qualitative approaches specifically, suggests that clinician experience is currently defined through self-reported information about what happened in the course of clinical work and how this impacted a clinician. The absence of validated questions and survey tools regarding clinician experience creates a methodological challenge for researchers and health services. Few studies utilised direct observation or structured surveys to document what happened across a given period or process. The inclusion of work-related measures, particularly regarding engagement, work environment, culture and job satisfaction when capturing experience data suggests that such factors are important in the context of clinician experience. Psychological research highlights across multiple sectors the association between workforce engagement, retention and organisational commitment, with higher levels of engagement also associated with job satisfaction, health and performance [71–73].

The complexity of the construct of experience is reflected in limited understanding through research relating to the concept of clinician experience and how this can be measured. Yet, knowledge of the factors that contribute to experience and of what an optimal experience outcome is in the context of clinical care, is required to both measure and apply the information to enhance clinician experience [6]. Features of the work environment appear to be indicators of a positive or negative clinician experience, with factors such as leadership, work scheduling, professional relationships, management support and safety culture identified across multiple articles [34, 37, 43]. Assessment of the work environment in the context of understanding experience is therefore important. Further work to delineate the relationships between these features of the work environment, clinician experience and outcomes of care would be valuable.

Survey tools were utilized in 24 of the identified articles. Where surveys were used to assess clinician experience in relation to health systems change or performance, surveys generally comprised of researcher-developed items that did not undergo validation about clinician experience. Instead, this research was accompanied by validated tools capturing data of work environment, satisfaction, engagement or psychological outcomes [28, 29, 47, 48, 50, 61]. The limited application of survey tools and the need for researcher-developed components, reflects the lack of clarity regarding definition of clinician experience and the limited availability of existing validated tools in this area as a result. The Picker Staff/Employee Questionnaire was the only tool that emerged that attempted to provide a validated instrument for capturing experience data amongst clinicians. The two articles in which this was used reported different adaptations of the Picker Questionnaire and only one of these by Stahl et al. (2017) provided validation data [57, 60]. The nuanced nature of experience lends itself to qualitative research methods, reflected in the high volume of qualitative research articles identified [74].

Clinician experience data have been utilised for the implementation and evaluation of change projects in health services and systems in many countries. A key purpose of clinician experience data is the provision of information from clinicians about factors that encouraged or enabled them to adopt, adhere or adapt to new practices, technologies and circumstances. Most studies identified in the peer-reviewed and grey literature were focused on clinician experiences of change related to a specific event or project and clinicians were viewed as critical to ensuring that proposed health service or system changes was taken up in practice so that benefits could be realised [19, 23, 25, 75, 76].

### Implications

Little is known about how experiences of providing care generally impact clinicians and health system change. Data in the articles were used primarily to provide understanding of the experience of undertaking clinical work in changing contexts [57, 70]. Patient experience has been explored in terms of key features of the healthcare process and of specific healthcare events [77]. In the context of clinician experience, our findings suggest that health systems may seek to further develop data collection instruments that identify key indicators of positive clinician experience that have identified links to retention, well-being and performance, such as clinician engagement which has received substantial attention [78–80]. A more developed understanding of the key indicators of clinician experience that are impacted by health system changes would be valuable towards developing measures that capture the effects of health system change on the experience of providing care. In health system application, a broader ‘clinician pulse’ style assessment may be utilised by organisations to compliment this information and understand the experience of clinical work on a continuum rather than in the context of episodes of change or care.

### Limitations

This rapid review of evidence was limited to articles from countries with broadly comparable health systems which include the Organisation for Economic Co-operation and Development (OECD) countries. Applying rapid review methodology also limited the scope of data sources explored which was necessary in the context of an expansive literature but may have led to the omission of relevant material. The limited definition of clinician experience may have also led to the omission of relevant material that utilised alternative terminology.

## Conclusion

Knowledge of clinicians’ experiences of providing care in clinical contexts is lacking, and studies have focused on capturing the subjective experiences of clinicians involved in episodes of change. Some health systems have sought to capture clinician experience data via staff surveys and these data are not clearly distinguished from data of the work environment, engagement, and psychological experiences associated with clinical work. Clinician experience data appear to be primarily used to provide an overview of how clinicians are feeling about the service or system in which they work and to enhance understanding rather than to create change. The assessment of clinician experience may be captured through adapted and existing staff survey tools, but psychometric and content analysis is required. Moreover, more evidence is needed of these existing service or system-level survey tools to health service or system enhancement. Evidence indicates that clinician experience data has been utilised for understanding barriers to the adoption of changes and approaches that clinician’s take to adapt to change in their environment. In progressing the value-based healthcare agenda, health departments may wish to consider the indicators of positive clinician experience as a basis for exploring how to optimise clinician experience.

## Supplementary information

**Additional file 1.**

**Additional file 2.**

**Additional file 3.**

## Data Availability

Not applicable.

## Abbreviations

OECD: Organization for Economic Co-operation and Development
PES–NWI: Practice Environment Scale–Nursing Work Index
NWI: Nursing Work Index
NHS: National Health Service
NSW: New South Wales
REA: Rapid evidence assessment
UK: United Kingdom
VBHC: Value-based healthcare
