# Supplementary material for: Clinicians’ experience of providing care: a rapid review
Source: BMC Health Serv Res. 2020 Oct 15;20:952. doi: 10.1186/s12913-020-05812-3 (PMC7559170; doi:10.1186/s12913-020-05812-3)
Supplement: Supplementary file 1 — Additional file 1. [file 12913_2020_5812_MOESM1_ESM.docx]

**Appendix 1**

The websites of the following organisations were searched to identify relevant work, publications and/or reports into clinician experience of care delivery. Comparable health systems such as England, Canada, New Zealand and Northern Europe; generally not the US.

Focused to the concepts of ‘changing’ and ‘new’.

Key search terms: chang*, transform*, clinician engagement, implement*, introduce*, clinician experience, ways of working, disrupt*, models of care.

Key sites for each country include:

- Quality and Safety Commissions
- Departments/Ministries of Health
- Professional bodies, e.g. medical colleges
- Other health organisations (govt or NGO) that support healthcare quality and safety initiatives or commission reports.

1. Australian Commission on Safety and Quality in Health Care (Australia)
2. Department of Health (including state DoH/MoH websites) (Australia)

- NSW Health Agency for Clinical Innovation (Australia)
- NSW Health Clinical Excellence Commission (Australia)
- Safer Care Victoria (Australia)
- Clinical Excellence Queensland (Australia)

1. Australian College of Nursing (Australia)
2. Royal Australasian College of Physicians (Australia and New Zealand)
3. Health Quality & Safety Commission (New Zealand)
4. Ministry of Health (New Zealand)
5. Ko Awatea Health System Innovation and Improvement (New Zealand)
6. Health Canada (including provincial websites) (Canada)
7. Royal College of Physicians and Surgeons (Canada)
8. Canadian Foundation for Healthcare Improvement (Canada)
9. Care Quality Commission (UK)
10. National Health Service (including NHS England, NHS Scotland and NHS Wales) (UK)

- NHS Improvement (UK)

1. Royal Colleges of Physicians, Surgeons, Nursing (UK)
2. The Health Foundation (UK)
3. International Society for Quality in Healthcare
4. World Health Organisation (particularly Regional Office for Europe)
5. European Society for Quality in Healthcare
6. Institute for Healthcare Improvement (US)

(particularly the IHI Health Improvement Alliance Europe)

- Platform for Continuous Improvement of Quality of Care and Patient Safety (PAQS) (Belgium)
- Danish Society for Patient Safety (Denmark)
- The Kings Fund (UK)
- STZ Hospitals (The Netherlands)
- Qukturum, Jonkoping (Sweden)

[Additional European websites]

1. Ministry of Health (Denmark)
2. Healthcare Denmark (Denmark)
3. The National Board of Health and Welfare (Sweden)
4. Ministry of Health and Social Affairs (Sweden)
5. Ministry of Health and Care Services (Norway)
