# Supplementary material for: Clinicians’ experience of providing care: a rapid review
Source: BMC Health Serv Res. 2020 Oct 15;20:952. doi: 10.1186/s12913-020-05812-3 (PMC7559170; doi:10.1186/s12913-020-05812-3)
Supplement: Supplementary file 2 — Additional file 2. [file 12913_2020_5812_MOESM2_ESM.docx]

Appendix 2: Summary of included studies from electronic database search

| **Authors** | **Year** | **Method/**  **System where clinical care is provided** | **Aims/Objectives** | **Setting and country** | **Method of data collection and Sample** | **Key findings (relating to methods of clinician experience of delivering care)** |
| --- | --- | --- | --- | --- | --- | --- |
| Abad-Corpa, E. et al | 2013 | Implement Evidence based Clinical Practice (EBCP) (participatory action research)-PAR | To gain a better understanding of the experience of change | Tertiary level general university hospital  Spain | - Qualitative, multi - Six nurses - Five group meetings - Individual reflections in participants’ dairies. - Participant observation | - PAR was adopted to implement EBCP - Nursing staff were invited through a project presentation; initially this started with 40 nurses but reduced to 6 nurses because only they were able to participate in all the sessions planned for this project. - An initial needs assessment was carried out about the current clinical practice (CP) in the unit which showed that enormous variability in CP which had repercussions for the quality of care and the healthcare results of the patients - The variability was related to training deficit and limited institutional support and resources. - Participant perceived that using evidence would standardize practice to work better in teams, improving both confidence and professional recognition. - The changes proposed in terms of clinical practice guidelines were more pragmatic for the PAR group who acted as a ‘user’ of evidence - The PAR group began to self-organize and began to revise the evidence of important clinical topics. - The nurses/ participants were leading implementing the changes- this is what aided in bringing the change in CP |
| Abrahamsen et al. | 2017 | Introduction of a new (Orthogeriatric) unit | To gain understanding of clinicians’ experience of delivering care with an interprofessional group | Hospital Denmark | - Qualitative - Four focus groups interviews with 19 healthcare workers | - The introduction of the new unit took place and the experience of the clinicians (including geriatricians, orthopedic surgeon, nurses, nurse assistant, occupational therapist and physiotherapist) was explored post-two years. - Three main themes: holistic-patient centered care, professional growth and interprofessional collaboration. - Allowed to deal with more complex issues - This arrangement also posed challenges and more stress for nursing staff and nursing assistants because of work overload. They also had feeling of inadequacy |
| Alami, H et al | 2017 | Eastern Quebec Telepathology Network (EQTN) | The aim was to evaluate EQTN in order to identify and analyze the factors and issues associated with its implementation and deployment, as well as those related to its sustainability and expansion. | Hospitals  Canada | - Qualitative, multi - Interviews, focus groups, and discussions with 9 clinicians (pathologists and surgeons) and other stakeholders   (including decision-makers, clinical and administrative project managers, and technologists) | - in this case, telepathology helped curtail pathologist travel to several locations, a phenomenon which has resulted in clinical time gains. - Clinicians though that telepathology could also be relevant for other clinical activities and other specialties (eg, endoscopy, gynecology, and orthopedics). - The use of technology has influenced an evolution of scientific evidence and changes in clinical protocols. - The clinical change implemented has changed the retention and recruitment of doctors in rural sites for example: Telepathology was found to be of little help when it comes to promoting the recruitment and retention of pathologists in remote areas. On the other hand, telepathology has enabled recruiting and retaining surgeons in certain locations. - New roles have gained importance in the provision of care. For example: Technologists have seen their work evolve and have to some extent become “pathologists’ assistants.” However, this status is not yet recognized in Quebec. |
| Amann et al | 2018 | adopted a pragmatic epistemological approach | This study explored healthcare professionals’ accounts of patient participation, focusing particularly on aspects related to patients’ contributions to the planning and design of healthcare services and products. | Hospitals  (four specialized centers for spinal cord injury)  Switzerland | - Qualitative - Semi-structured interviews with healthcare professionals | - Participants referred to three types of patient contributions that would usually emerge from informal exchange: (1) bringing in information unknown to staff; (2) reporting problems; and (3) providing concrete suggestions for improvement. - Patient participation was favorably viewed by the clinicians - Participants reported that patients provide feedback on the healthcare organization, by identifying problems and making formal or informal complaints |
| Auta et al | 2015 | Extended role of pharmacist in prescribing medications | This study investigated the facilitators to change in hospital pharmacy practice in England in order to identify lessons that might assist in the potential changes needed in other countries for extended clinical roles. | Hospital pharmacy practice in England | - Qualitative - Semi-structured interviews was conducted with 28 participants, comprising 22 pharmacists and 6 pharmacy technicians | - New role of the pharmacist is introduced which has improved efficiency of health care provision. - The new role also needs a change in the educational systems for example in this case: participants reported about the changes in education and training of pharmacists. These changes including the introduction of the postgraduate clinical pharmacy programmes to address many of the shortcomings of pharmacists’ clinical knowledge and skills as a result of the expansion of pharmacy services into clinical areas. - In the beginning the changing roles faced challenges but many also supported this new role. - strategies were identified to facilitate pharmacists’ new roles- These included freeing-up pharmacists’ time for clinical roles |
| Baathe F, Rosta J, Bringedal B, et al. | 2019 | Clinicians perceptions about professional fulfilment, organizational factors and quality of patient care. | The aim of this study was to explore how doctors experience the interactions among professional fulfilment, organizational factors and quality of patient care. | Surgical department of a mid-sized hospital  France | - Qualitative - Seven exploratory, semi-structured interviews with doctors (representing approximately 30% of the population). - A feedback session was held with rest of the doctors to confirm the findings of this study. | - Clinicians describe ‘stretching themselves’ in order to provide quality care to the patients, that is, handling the tensions between quantity and quality and to overcome organizational shortcomings. - Experiencing a workplace emphasis on production numbers and budget concerns led to feelings of estrangement among the doctors. - Clinicians reported a shift from serving as trustworthy, autonomous professionals to becoming production workers, where professional identity was threatened. They felt less aligned with workplace values, in addition to experiencing limited management recognition for quality of patient care. - The clinicians value patient care and thus individually develop initiatives to facilitate their workflow. |
| Baik and Zierler | 2019 | purposeful interprofessional (IP) team intervention in practice | To aim was to explore clinical nurses’ experiences and perceptions following a purposeful interprofessional (IP) team intervention in practice. | Academic medical hospital.  USA | - Qualitative study - Exploration of using focus group interviews of registered nurses (n=10) who care for patients with advanced heart failure. | - During the intervention there was improved communication (in terms of clarity and accuracy) between staff members which helped better understand roles. This experience then impacted how care was provided. Although there were reports of no improvement when dealing with attending physicians. - More openness in the work environment. - IP intervention improved quality of patient care. - Improved job satisfaction because of improved teamworking and communication |
| Bayes et al | 2018 | Introduction of the best available evidence into health care practice  Overall model was Glaserian Grounded Theory methodology. | The aims of this study were to explore Australian change-leader midwives’ experiences of implementing evidence-based innovations, the factors that contribute to evidence based clinical practice or process change implementation success or failure in midwifery practice settings. | midwifery practice contexts  Australia | - Qualitative - Semi-structured interviews - n=16 | - The midwives change in practice initiatives were explored. - Midwives reported that initiating change, whether requested to by the service they work in or on their own, face myriad challenges from inception to the end of the process. - Bringing a change in practice is often faced with mistrust, opposition and resistance. - There is a lack of resources and broader organizational support when introducing the change |
| Bellagamba et al | 2016 | five departments (304 workers) relocation (medical, paramedical, administrative and technical staff) between two public health sites | This aim of this study is to compare quality of work-life factors between a relocated work group and a control group. | Five departments relocated between two sites of a public university hospital  France. | - Surveys and qualitative interviews, multi - Pre and post-test analysis with a comparison control group - The interviews (n=22) focused on the organization of the service, the working environment, and career plans. | - The survey explored the workers’ psychosocial job characteristics, their perceived health, and psycho-organizational constraints. - Survey:   - 51 ad-hoc questions exploring socio-professional characteristics and perceptions of working conditions   - 26 items of the Karasek Job Content Questionnaire (JCQ), which describes the ‘‘job strain’’ and ‘‘isostrain’’ associated with psychological demand, decision latitude, and social support (SS).   - 12 questions measuring the perceived mental health (MCS ‘‘Mental Component Summary’’) and perceived physical health (PCS ‘‘Physical Component Summary’’)   - 22 issues validated in French from Nursing Work Index-Extended Organization (NWI-EO). These questions explored the psycho-organizational constraints that describe the health care givers work environment - One third staff volunteered for the transfer - Most staff were satisfaction with their new location and had sufficient autonomy - Challenges were also present in terms of increased workload and stress which was make them consider to leave the job altogether. They also experienced deteriorated coworker relationships. |
| Bennetts et al, | 2012 | current pain management practice in Australian Emergency Department (ED) | To explore current pain management practice in Australian EDs and identify enablers and barriers for best-practice pain management. | Hospital  (Emergency department)  Australia | - Qualitative, multi - Five focus groups and two in-depth interviews were held with ED clinical staff (n = 47) from six hospitals in three states. | - Emergency department staff identified a gap between evidence-based pain management recommendations and everyday practice. - Perceived barriers to improving pain management (care provision): a lack of time and resources, a greater number of urgent and serious presentations that place pain management as a lower priority, organizational protocols and legislative issues. All groups noted difficulty in applying pain management guidelines in the context of competing priorities in the challenging ED environment. - Enablers: A culture of learning clinical practice should be driven from respected senior staff and peers - Participants expressed the view that evidence-based practice improvement should be championed by senior clinical staff, and that evidence to demonstrate the benefits of change must be presented to support the need for change. |
| Bloom and Huntington | 2010 | Electronic Health records (EHR) Implementation on clinical practice | The objective was to explore how faculty, residents, and both clinical and nonclinical staff view the effects of EHR implementation on a broad range of issues such as amount of time spent documenting and occurrence of documentation, effect on patient care, interference with other activities, effect on communication and relationships, coding/billing process, and overall efficiency | Primary care/family medicine  USA | - Quantitative- Survey at 8 months and 12 months post HER implementation | - Perception of faculty, residents, and clinic staff did not observe a benefit of the EHR system to patient care - Initially physicians were spending approximately 16 minutes for documentation and this reduced to 13 minutes. Physicians and residents are very dissatisfied with the amount of time required for documentation using the EHR system. |
| Bogh et al | 2018 | Impact of hospital accreditation (DDKM) on clinician experience | The aim of this study was to explore how staff understand hospital accreditation and its relation to quality improvement | Newly accredited hospital  Denmark | - Qualitative - Semi-structured interviews with staff members: doctors, nurses, quality coordinators | - Initially the DDKM was unclear and with time it began to make more sense - During the DDKM, clinicians were expected to spend more time on administrative tasks rather than patient care - During the DDKM and online library was established-this led to improved patient care levels despite staff turnovers. They were also using this library to seek information about care |
| Bove et al | 2018 | palliative outpatients’ structure for patients with COPD | The aim of this study was to explore the health professionals’ expectations and experiences of a new palliative out-patients structure for patients with advanced COPD | Hospital  (new outpatient organization (CAPTAIN))  Denmark | - Qualitative - Focus groups and individual interviews were conducted with pulmonary nurses, pulmonary doctors and municipality nurses | - The overall methodology was interpretive description (ID) an inductive approach that focuses on meaning and how generated knowledge apply to clinical practice. - Qualitative data from the pre-CAPTAIN phase was mostly concerns of the clinicians about the new program, the competency to use it and how it was to be delivered to the patients. There were many aspirations for how this new program will encourage clinical practice. - Post-CAPTAIN phase- the clinicians thought that this was a positive change for patients, but this triggered new concerns. Although the new program provided continuity of care and better understanding of the patient’s everyday life, their perceived values and preferences. Meanwhile this increased insight in the patients’ life and the awareness of suffering added an element of stress on the clinicians. In this phase new roles emerged for the clinicians; for example: new role moved from being highly specialized toward a general pulmonologist more similar to a GP. Also this program encouraged effective interprofessional collaborative work |
| Burau and Bro | 2015 | two different discharge models: add-on’ model and ‘embedded model | This study presents the case study of the introduction and routinization of explicit discharge arrangements for patients with prostate cancer in two hospitals in | Hospital  Denmark | - Qualitative - 12 focus groups with doctors, nurses and secretaries | - Clinicians acknowledged the specific advantages of the new discharge arrangements for the department at large (reducing the number of control appointments and, thereby, freeing resources for other patients). - Clinicians distanced themselves from the streamlined process, however they kept control of the decision about who to discharge through their individual professional judgment. - Engagement of the clinicians in this program greatly varied. |
| Burau and Overgaard | 2015 | Organizational change: introduction of case load midwifery | This study focuses on midwives’ role in introducing and developing caseload midwifery. | Three hospitals  Denmark | - Qualitative - Individual and group interviews - Participants included: caseload midwives, ward midwives, obstetricians and health visitors, management by chief midwives and their deputies. | - The new caseload organization facilitated their role in providing the care. The midwives pursued both professional and organizational interests in the new work environment. - The new model had midwives’ interest because it allowed a good work-life balance enabling them to combine work with family responsibilities - This model also provided an opportunity for professional development - Increased professional satisfaction because it allowed midwives to provide holistic and continued care to their patients. - On the other hand, some midwives felt that the quality of work and their personal work satisfaction were challenged by the frequent and long duty turns |
| Burney et al | 2012 | Needs assessment of treatment of sepsis in the emergency department | This study explored specific barriers to maximize benefits of a planned sepsis treatment initiative. A baseline assessment was conducted for knowledge, attitudes, and behaviors regarding detection and treatment of severe sepsis from clinicians | Hospital  USA | - Quantitative Survey - The survey explored:   (1) baseline knowledge and self-reported confidence in identification of systemic inflammatory response syndrome and sepsis;  (2) current practices in treatment;  (3) difficulties encountered in managing sepsis cases;  (4) perceived barriers to implementation of a clinical pathway based on early quantitative resuscitation goals; and  (5) to elicit suggestions for improvement of sepsis treatment within the department. | - N= 101 clinicians including staff and nurses completed a questionnaire which was designed to assess (1) baseline knowledge and self-reported confidence in identification of systemic inflammatory response syndrome and sepsis; (2) current practices in treatment; (3) difficulties encountered in managing sepsis cases; (4) perceived barriers to implementation of a clinical pathway based on early quantitative resuscitation goals; and (5) to elicit suggestions for improvement of sepsis treatment within the department. - Different barriers identified: delay in diagnosis, interprofessional work, organizational support in terms of access to equipment and space - Discrepancy in familiarity with the criteria of sepsis. |
| Chapman et al | 2011 | Needs assessment to explore reluctance to adopt guidelines | This study was conducted to explore clinicians’ attitudes and the clinical environment in which they work to understand their reluctance to adopt VTE prophylaxis guidelines | Two metropolitan hospitals  Australia | - Qualitative - Semi structured, open ended questions | - interviews revealed that barriers to evidence based practice include i) the fragmented system of care delivery where multiple members of teams and multiple teams are responsible for each patient’s care; ii) the culture of practice where team practice is tailored to that of the team head. - Participants described how senior clinicians practice   an ‘art of medicine’ which is the combination of medical knowledge, intuition, experience and judgement. That is Clinicians develop preferences or an ‘art’ towards patient management and treatment medicine which is considered to take precedence over guidelines. |
| Chouliara et al | 2014 | Implementation of a evidence based stroke early supported discharge services | To explore the perspectives of healthcare professionals with stroke Early Supported Discharge service in relation to: (1) the factors that facilitate or impede the implementation of the service, and (2) the impact of the service. | Hospital  (Discharge services)    UK | - Qualitative - Semi-structured interviews | - Facilitators of this program: (1) the adaptability of the intervention to the healthcare context, (2) the role of rehabilitation assistants and (3) cross-service working arrangements. - Perceived challenges included: (1) lack of clarity regarding the referral decision making process, (2) delays in securing social care input and (3) lack of appropriate follow on services in the region. - perceived impact of the services was: (1) reducing in hospital stay, (2) aiding the seamless transfer of care from hospital to the community and (3) providing intensive stroke specific therapy. |
| Chua et al | 2019 | Escalating care for clinically deteriorating patients in General wards | The objective of this study was to explore the experiences of junior doctors and nurses in escalating care for clinically deteriorating patients in general wards | Emergency Department teams (METs) in Changi General Hospital  Singapore | - Qualitative - Twenty-four individual interviews were conducted with 10 junior doctors and 14 registered nurses | - The decision to call a MET or the primary team doctors is a complex judgement - Participants' decisions to call the MET or to escalate to the primary team doctors depends on the severity of a patient's deterioration and perceptions of the primary team doctors' capacity to manage the patient. - Both doctors and nurses also reported that the fear of criticism from others above them in the medical hierarchy if they were perceived to have activated the MET “unnecessarily”. - Nurses reported the use of ISBAR (Identify, Situation, Background, Assessment and Recommendation) in their communication of deterioration to ward doctors. - Nurses face challenges in obtaining medical reviews from junior doctors for patients with deterioration - Nurses were more inclined to call the MET after office hours because on call doctors lacked an understanding of the deteriorating patient's condition |
| Cooper et al | 2013 | Action research approach to enable staff on rehab ward for older patients to engage in change activities. | To explore the facilitating factors that enabled staff on a rehabilitation ward for older people engage in change activities | Hospital  (Rehabilitation wards)  UK | - Qualitative - In-depth interviews with staff and managers | - Findings related to improvements in rehabilitation care arising from implementation of the action plan together with the facilitating factors that it was perceived enabled such changes and improvements to take place - Staff members felt standards of care had improved on the ward with the patient allocation system being seen as a contributory factor- improved relationship, more continuity of care and more patient focused - increased engagement with rehabilitation was also reflected in the involvement of staff and patients in social activities. - Staff identified that this work gave them a voice and that they felt valued and cared for as a result of someone listening to and showing an interest in them and their work. - The presence of the researcher was seen as a catalyst that ‘got the ball rolling’ through helping staff to highlight problems, come up with possible solutions and get them started. - Staff felt their knowledge and understanding was improved through formal and informal conversations and discussions that took place, work on change activities and informal prompting and coaching by the researcher when present on the ward. - Motivation for staff to improve practice was also increased through the achievements they made along the way. All staff were proud of the part they had played in helping change happen and care standards to improve, together with the recognition their work was receiving. |
| Cotta et al | 2015 | Implementing antimicrobial stewardship | To explore organizational factors and barriers contributing to limited uptake of antimicrobial stewardship (AMS) in Australian private hospitals and to determine solutions for AMS implementation. | Private hospital system  Australia | - Qualitative study - series of focus group discussions with a semi-structured guide | - consultant specialists practised with significantly more autonomy in private hospitals than in public hospitals because they were practicing as individual contractors. Also, it was difficult to expect a specialist to be accountable to anyone else in this environment - private hospital lacked ability to influence antimicrobial prescribing. - most private hospitals viewed consultant specialists as their ‘customers’ and so were apprehensive about enforcing guidelines and hospital policies and procedures. - It was reported that many specialists wanted to know what their colleagues were prescribing and whether they were consistent with the practice of others- this could be used, in the form of perceived peer pressure, to promote uniformity in clinical practice - Referral to ID physicians- lack of ID physicians and Consultant specialists were willing to refer patients to ID physicians when appropriate - Barriers and potential solutions were discussed in relation to the implementation of how AMS could be implemented |
| Creswick et al | 2011 | Implementation of PACS picture archive and communication systems | This study examined whether and how ICU nurses view and use images and whether access to PACS promotes innovation in work practices. | ICUs at 3 metropolitan teaching hospitals  Australia | - Qualitative, multi - Interview and observation of ICU nurses | - PACS to ICU settings promotes changes in nursing work practices by providing nurses with the ability to act more autonomously - spending less time searching for x-rays, and that the turnaround time for the availability of images for viewing had decreased. - Nurses reported viewing images at the start of their shifts, especially for intubated patients who routinely receive a chest x-ray early each morning, and later in the day if required. |
| Dainty et al | 2013 | Implementation of a large-scale QI | The aim of this study is to understand ICU staff perspectives on collaborative QI based involvement in a multiorganizational improvement network and its impact on providing care | Community hospitals ICUs  Canada | - Qualitative - Key informant interviews were conducted with staff from 12 community hospital ICUs | - clinicians reported that belonging to a collaborative network provided recognition for the high-quality patient care that they already provided. It also was form of feedback to how well they were performing compared to other ICU participating sites. - QI collaborative networks promote behavior change by improving intrateam communication, |
| Dellve et al | 2018 | Organizational redesign of care processes | This study focuses on how work conditions contribute to these different aspects of engagement including clinical engagement behaviour (during organizational redesign of care processes among different groups of healthcare clinicians) | 5 hospitals  Sweden | - Quantitative- Survey - Surveys were distributed at the start and then one year | - In this context of healthcare clinicians' engagement in organizational improvements:   - engagement mean attitudes toward engagement in organizational development,   - work engagement as a cognitive state,   - clinical engagement behavior in developing patient safety and quality of care in practice. - Clinical engagement behavior assessment: Two scales were used to assess clinical engagement in patient safety and quality of care - There were associations between positive attitudes toward organizational improvements and stronger clinical engagement in developing quality of care (r = 0.37, p < 0.00) and patient safety (r = 0.34, p < 0.00, respectively). Negative attitudes were associated with decreased clinical engagement behavior. |
| Dix et al | 2012 | Intentional rounding for nursing staff | This study discusses the roll out of intentional rounds and its impact on providing care | Hospital  UK | - Audit and Quantitative survey - Audit of the number and frequency of call bells at the same time but different days of the week. | - Reduced frequency of call bells - Early identification of pressure ulcers - Increased patient satisfaction - In terms of staff perception there was reduced time spent with the patient - Staff could not spend more time with patients who needed more attention - Staff were less satisfied with the new arrangement |
| Draper | 2018 | transition from student to newly qualified nurse (NQN) | To explore the experiences of NQNs also employed as health care assistants (HCAs) during their pre-registration education programme and how this prior and ongoing HCA experience influenced their transition experiences. | Health system  UK | - Qualitative - Telephone interviews with participants | - Participants frequently talked about the increased responsibility of their new role. This heightened sense of responsibility also encouraged them to ask questions if they needed help or to question practice - There were raised expectation of others around them and how they were perceived by patients and relatives as a person in a position of authority - realization that being a qualified nurse was more complex and busier than previously imagined. - Management of identities previous and new - The change in attitude towards them as a result of this change in uniform came as a surprise - Informal support was provided by family, friends (some of whom were also nurses) and by workplace colleagues, while Formal support structures included preceptorship, |
| Elliot et al | 2016 | new format of charts for recording observations and as a prompt for responding to episodes of clinical deterioration in adult medical–surgical patients | To examine user acceptance with a new format of charts for recording observations | Hospital  Australia | - Quantitative and qualitative Survey - Surveys were had open-ended comments and narrative from short informal feedback groups providing elaboration and context of user experiences. | - 28-item survey was developed to examine staff perceptions and experiences with the design and content of the chart for usability in the clinical setting: clarity of text, layout, completeness, ease of documenting and utility in prompting a response for a deteriorating patient - Easy to use but does not fit patient file - The ‘modifications to calling criteria’ section was most frequently commented on during handover debriefings - Respondents reported that the charts assisted in identifying a patient at risk , aided management of the deteriorating patient and enabled effective clinical handover of the patient’s condition |
| Foster et al | 2016 | Service delivery innovation | This study explores the perspectives of clinicians and managers involved in a general practitioner-led integrated diabetes care innovation | GP-led integrated diabetes care in primary health care  Australia | - Qualitative, multi - Focus groups and semi-structured interviews at two primary health care sites | - There were three main themes: 1) trusting and embedding new professional relationships; 2) synchronizing services and resources; and 3) reconciling realities of innovation work - there was a change in the traditional way of thinking about diabetes care and professional roles. - There was reluctance among specialists for GPs to provide care because of the negative experiences with GPs delivering less than expected follow up care for patients. - It was important to take steps to improve communication with patients’ regular GPs (i.e. peer-to-peer communication between the model’s clinical fellows and referring GPs was critical) - added benefits of earlier access for patients and reduced waiting lists. - Good communication and information sharing about patient care was critical. |
| Fowler et al | 2018 | Patient access to primary care | This study aims to explore general practitioners’ (GPs’) views and experiences of an Enhanced Primary Care programme (EPCP) which works to extend patient access to primary care. | Primary care practice, Sheffield  UK | - Qualitative - Semi structured interviews with GPs across 24 practices | - GP view were variable about their acceptance to the EPCP and this was due to their view about the role of GP practice. - Some viewed EPCP program as a short-term centrally driven initiative while others saw it as a pragmatic solution to manage additional demand. - GPs were skeptical about the use of additional appointments for non-urgent cases and questioned their relative benefit to recipients of care. - Financial imperatives were key drivers for involvement in the program. GPs saw the opportunity to use incentive payments within the EPCP as additional funding for their practice. - Most GPs indicated limited awareness of most of the schemes in relation to additional services and professionals, which were intended to provide new ways of working. - Some GPs who evidently needed to manage capacity and demand within the finite resources and workforce reflected on the opportunities afforded by additional out of hours (OOH) clinics. - GPs also questioned the acceptability of a centrally delivered scheme to patients who are best served by ensuring continuity of care. |
| Haynes et al | 2011 | implementation of a checklist-based surgical safety intervention | To assess the relationship between changes in clinician attitude and changes in postoperative outcomes following a checklist-based surgical safety intervention | 8 Hospitals  USA | - Quantitative Survey - Pre- and post-intervention survey with clinical staff working in the operating room | - The survey used is a Modified operating-room version Safety Attitudes Questionnaire (SAQ). - Main outcome measures include: Change in mean safety attitude score and correlation between change in safety attitude score and change in postoperative outcomes, plus clinician opinion of checklist efficacy and usability. - The degree of improvement in mean SAQ score at each site correlated with a reduction in postoperative complication rate - The checklist was considered easy to use, some thought it took a long time to complete, and others felt that the program prevented errors. Overall, majority would want the checklist used if they were undergoing operation |
| Høstgaard et al | 2017 | Constructive eHealth evaluation method (CeHEM) | The aim of this study is to explore the introduction of electronic health records (EHR) | 4 hospitals  Denmark. | - Qualitative and Quantitative, multi - Observations in wards - Interviews - Document access - Surveys survey of the clinicians’ assessment of the clinical benefits of the new EHR after implementation. | - The main interest of the clinicians (and of the physicians in particular) was the clinical benefits and high user-friendliness. - the new EHR improved patient health and safety and the quality of treatment - some physicians indicated that they did not use the system at all because they felt it hampered their clinical work. |
| Huby et al | 2014 | This article uses theories of social capital to understand ways in which negotiation of professional boundaries among healthcare professionals relates to health services change. | This study explored how negotiation of professional boundaries among healthcare professionals relates to health services change. | Primary care organizations (PCOs) in UK | - Qualitative - Serial Interviews with key clinicians as they progressed with collaboration, negotiation and contesting developments. | - a great deal of explicit expansion and consolidation of professional and organizational territory which depended on collaboration that was to everybody’s advantage. - professionals’ work to protect and expand their claims to work territory, - remuneration and influence is a catalyst for development and was also necessary to establish professional boundaries that underpinned novel service arrangements; for example: The new arrangements created a more business-like approach to the establishment, monitoring and running of services. Services previously based on informal collaboration and with no budget came under threat. Existing services had to be redefined by their cost and income - Conflict and contest was less of a threat to change, however, a lack of engagement in boundary work brought change because this engagement produced relationships based on shifting professional allegiances across and along boundaries, and these relationships mediated the social capital needed to accomplish change. |
| Irvin et al | 2013 | Oncology Nursing Society (ONS) and ONS Foundation worked together to develop the Institute for Evidence-Based Practice Change (IEBPC) program to facilitate the implementation of evidence-based practice (EBP) change in nursing | This study explored the experience of 19 teams of nurses from various healthcare settings who participated in the IEBPC program | Hospitals  USA | - Qualitative and observations - Qualitative analysis of verbatim narratives of activities and observations during the process of implementing an EBP project | - EBP implementation enabled participants to learn about their own practice and to experience empowerment through the evidence, and it ignited the spirit of inquiry, team work, and multidisciplinary collaboration. - Several teams incorporated recognition and the use of incentives into project implementation. These strategies aimed to get staff attention and encourage participation in project activities and related changes to patient care. - Conversely, lack of engagement of key groups inhibited progress. Inadequate communication inhibited progress, and teams noted that good communication was a critical success factor. - Areas of discovery expressed in narratives included learning about their practices, learning about each other, igniting the spirit of inquiry, experiencing empowerment through evidence, and learning about the challenge of sustaining improvement. |
| Jeffs et al | 2015 | Implementation of Antimicrobial Stewardship at 3 Academic Hospitals | To assess the perceptions and experiences of antimicrobial stewardship program leaders in terms of clinicians’ attitudes toward and behaviors related to antimicrobial prescribing | 3 academic hospitals  Canada | - Qualitative - Semi structured interviews were conducted with 6 antimicrobial stewards (2 physicians and 4 pharmacists) | - antimicrobial stewardship program engaged to get the right people on board throughout the organization - building relationships with clinicians in each ICU was also identified as a key process for the antimicrobial stewards. This occurred both formally (e.g., during rounds and face-to-face meetings) and informally (e.g., having coffee or hallway conversations). - During the implementation it was considered how best to overcome the possible reluctance and resistance of prescribers in the ICU to change their current antimicrobial practices so as to minimize the use of unnecessary broad spectrum antimicrobials and how best to influence the uptake of rational antimicrobial use. - Over time, the anti microbial stewards were able to demonstrate that antimicrobial use and positive patient outcomes were associated with a reduction in costs through prospective auditing of and feedback about antimicrobial prescribing practices. - The stewards were mindful and respectful when working with the ICU clinicians, tailoring the program according to each ICU’s culture and context. |
| Johansson et al | 2013 | experience of working on a locked acute psychiatric ward | This study explores the experience of health-care staff working on a locked, acute psychiatric ward. | Hospital  (Acute psychiatric ward)  Sweden | - Qualitative - Interviews with health-care staff (n = 10) | - health-care staff have to manage a changing and demanding work environment including a heavy and intense workload with limited opportunities for relaxation. - Positive experiences include: meaningfulness and personal development - The health-care staff described the importance of delivering good quality care - Health-care staff were engaged in ongoing changes to what should constitute the focus of care. For example: The staff said that it was the need for specialized nursing care, not primarily medical needs, that determined whether or not a patient should be admitted to the ward. - Nursing was adjusted to patients’ needs concerning - Clinicians wanted a stable management, continuity in patient contact and control of patients. - Knowing the patients was important for the health-care staff’s sense of security; they described how, when a new and unknown patient arrived on the ward, it felt safer to meet them together with a colleague - Clinicians felt a sense of responsibility- which was illustrated as a driving force in completing work tasks and caring for the patients’ well-being, but it could also lead to feelings of burden |
| Jones et al | 2013 | safety netting advice first contact clinicians give parents of acutely sick young children | The aim of this study is to understand what safety netting advice first contact clinicians give parents of acutely sick young children, how, when, and why. | General practice surgery, a District General Hospital emergency department, a paediatric emergency department, and an out-of-hours service  UK | - Qualitative, multi - Interviews and focus groups were held with doctors and nurses in a. | - They described that safety netting advice includes advising parents what to look for, when and where to seek help. - no participants described being trained in this area - Safety netting appeared to be rarely documented and was left to individual preference. - Participants described that safety netting was influenced by clinicians’ experience, confidence, time and knowledge; and perceived parental anxiety, experience, and competence. - Participants noted several limitations to safety netting including not knowing if it has been understood by parents or been effective; parental difficulty interpreting information and desire for face-to-face reassurance; and potential over-reassurance. |
| Kilpatrick et al | 2012 | boundary work following the introduction of an acute care nurse practitioner role in healthcare teams | This study aims to understand the process by which the boundaries between professions changed, following the introduction of an acute care nurse practitioner (ACNP) role and how this would affect the scope of practice and the team’s ability to give patient care | Two university-affiliated teaching hospitals in Canada | - Qualitative - Individual and/or focus group interviews and document analysis. | - The need to create space for the ACNP role was particularly salient for the team that was in place prior to the arrival of the ACNPs and this was done by making adjustment to own activities to integrate their role. - They talked about creating a psychological space or a mindset where the physicians could accept that ACNPs assumed some of their functions. - Participants described specific instances of lost role functions in the context of the professional groups following the introduction of the ACNP role in the team. - Participants described how experienced staff nurses lost status in the team and with the physician group because the physicians sought out the ACNPs for information. - As an early reaction to loss, the professional groups who   had experienced the most losses described a sense of mourning, a feeling of being abandoned and being pushed aside.   - Trust was believed to be the key to the successful completion of boundary work among team members and was enhanced between the ACNPs and team members as the ACNPs gained experience. - Some interpersonal dynamics improved the team dynamics with this new arrangement, while there was an overlap of many roles. |
| Kirkendall et al | 2013 | Transition to a full electronic health record (HER) | To examine healthcare worker’s perceptions, expectations, and experiences regarding how work processes, patient-related safety, and care were affected when a quaternary care center transitioned from one computerized provider order entry (CPOE) system to a full electronic health record (EHR). | Hospital  USA | - Quantitative Survey - Pre and post test using the Information Systems Expectations and Experiences (I-SEE) survey - The I-SEE survey was administered prior to and 1-year after transition in systems. | - The I-SEE contains 35 questions/items distributed across 7 scales: - Provider–patient communication (3 items) - Inter-provider communication (3 items) - Inter-organizational communication (2 items) - Work life changes (4 items) - Improved care (7 items) - Support and resources (8 items) - Patient care processes (8 items) - The majority of respondents were nurses and personnel working in the acute care setting. - Mean scores for each factor indicated that attitudes and expectations were mostly positive and score trends over time were positive or neutral. - Nurses generally had less positive attitudes about the transition than non-nursing respondents, although the difference diminished after implementation. |
| Lacasta Tintorer et al | 2018 | telemedicine systems available to facilitate communication between care levels between primary care (PC) and non-GP specialist care (SC) professionals - Online Communication Tool (ECOPIH platform) between Primary and Hospital Care was created | The objective of this article is to explore healthcare professionals’ views on communities of clinical practice (CoCPs) and the changes that need to be made after an online communication tool | Primary Care Service (PCS)  Spain | - Qualitative, mixed - Focus groups, triangular groups and individual interviews. | - For a system of communication between PC and SC to become a tool that is habitually used and very useful, the interviewees considered that it would have to be able to find quick, effective solutions to the queries raised, based on up-to-date information that is directly applicable to daily clinical practice. - Contact should be virtual – and probably collaborative – via a platform integrated into workstations and led by PC professionals. - Organisational changes should be implemented to enable users to have more time in their working day to spend on the tool. - It is also important to make certain technological changes, basically aimed at improving the tool’s accessibility, by integrating it into clinical workstations. |
| Langhan et al | 2015 | Implementation of newly adopted technology in acute care settings | This study explored experiences of acute care providers with the introduction of technology and identified barriers and facilitators in the implementation process. | Hospital  USA | - Qualitative - Individual interviews among a purposeful sample of 19 physicians and nurses within 10 emergency departments and intensive care units was performed | - Five major categories emerged:   - decision-making factors- adoption of new technologies was often varied and poorly understood by clinical staff; meanwhile Staff champions or early adopters helped to initiate the adoption of technology.   - the impact on practice- Participants mentioned how specific technologies on the unit are only used by certain providers or in certain patient populations, as compared to other technology that is used by all providers or is applied to all patients, such as electronic medical records or patient monitors.   - technology’s perceived value- Technology was perceived in a positive light because it was beneficial to providers, to patients or to trainees.   - facilitators to implementation. - Barriers included negative experiences, age, infrequent use and access difficulties. |
| Lelubre et al | 2018 | interprofessional medication adherence program (IMAP) for chronic patients | This study assesses the capacity of a physician and a nurse at the infectious diseases service of a public hospital and of community pharmacists to implement the IMAP in their practice. | Hospital  Switzerland | - Quantitative and qualitative analyses of the implementation process were conducted following the RE-AIM model (reach, effectiveness, adoption, implementation and maintenance | - Facilitators for reaching patients were their inclusion by the physician and the nurse and specifically the inclusion of naïve HIV patients, to whom the program was presented as a package linked to the new treatment. - The main reported reason for refusal was the patient’s reluctance to change pharmacy, especially because of an existing trust relationship with the pharmacist - Different barriers have been encountered by healthcare professionals to reach patients. These can be HIV-related difficulties (psychosocial issues with stigmatisation, denial and need for a high confidentiality level) and the limited number of trained pharmacies, reducing the choice for patients. The small population base of HIV patients also explained the small number of included patients. - All healthcare professionals agreed that the program was useful for patients. Firstly, they observed improvements in clinical results. Secondly, patients developed a relationship of trust with the pharmacist, who became a reference person for the patient at the pharmacy. Thirdly, the use of electronic pillboxes seemed to reassure patients, allowing them to visualize their medication intake. - The total time needed at the hospital to deliver the program was 30 to 40 min per patient - largest barrier encountered by healthcare professionals was a lack of time, related to lack of resources. For the physician and the nurse, the lack of time was a barrier to the program when a patient had somatic problems that had to be prioritized during the medical visit. |
| Leong et al | 2017 | Introduction of a perioperative briefing and debriefing | This study was carried out to improve patient safety in the operating theatre by the introduction of perioperative briefing and debriefing, which focused on an optimal collaboration between surgical team members. | Operating theatres of a tertiary care hospital Netherlands. | - Quantitative Survey - A prospective intervention study with one pretest and two post-test measurements: 1 month before and 4 months and 2.5 years after the implementation of perioperative briefing and debriefing | - The primary outcome was changes in the team climate, measured by the Team Climate Inventory. - Secondary outcomes were the experiences of surgical teams with perioperative briefing and debriefing, measured with a structured questionnaire, and - an independent observer observed the duration of the briefings - the team climate increased statistically significant (p≤0.05). - They perceived a higher efficiency of the surgical program with more operations starting on time and less unexpectedly long operation time. - The perioperative briefing took less than 4 min to conduct. |
| Leslie et al | 2017 | Implementation of Health information technology (HIT) | To identify the impact of a full suite of health information technology (HIT) on the relationships that support safety and quality among intensive care unit (ICU) clinicians. | three ICUs in three academic hospitals  US | - Ethnographic study and qualitative interviews | - Significant variation in HIT implementation rates and usage was noted. Average HITuse on the two “high-use” ICUs was 49 percent. On the “low-use” ICU, it was 10 percent. - Clinicians on the high-use ICUs experienced “silo” effects with potential safety and quality implications. HIT work was associated with spatial, data, and social silos that separated ICU clinicians from one another and their patients. Situational awareness, communication, and patient satisfaction were negatively affected by this siloing. |
| Li et al | 2012 | nurse practitioners plus adjunct information and communication technology for their new clinical role | This study aimed to investigate ways in which Nurse Practitioners (NP) have incorporated the use of Information and Communication Technology (ICT) as a mechanism to support their new clinical role within Emergency Departments. | Two teaching hospitals  Australia | - Qualitative - Semi-structured and in-depth interviews | - The purpose of the NP role was largely perceived by physicians as the alleviation of their sub-acute workload and expediting the treatment of patients of lower acuity. - ICT supported the advanced practice dimension of the NP role in the following ways: availability and completeness of electronic patient information enhanced timeliness and quality of diagnostic and therapeutic decision-making, expediting patient access to appropriate care. - improved quality of communication between health professionals within and across sites, with wider diffusion of the Electronic Medical Record holding the potential to further facilitate team-based, holistic care. |
| Liberati et al | 2015 | Introduction of patient centered model | The aim of this study is to understand how the introduction of a patient-centered model (PCM) in Italian hospitals affects the pre-existent configuration of clinical work and interacts with established intra/inter-professional relationships. | Hospital  Italy | - Qualitative - exploratory interview study and case study | - The introduction of the PCM challenges clinical work and professional relationships. - frontline clinicians believe that the new criteria for patients’ placement may disorient patients rather than improve their care process: clinicians suggest that patients and care givers’ main psychological need is to identify their care provider (the medical specialist) rather than to be placed in the “most adequate care setting” - The “political narrative” (the views conveyed by formal policies and senior managers) focuses on the power shifts and conflict between nurses and doctors, while the“workplace narrative” (the experiences of frontline clinicians) emphasises the problems linked to the disruption of previous discipline-based inter-professional groups. |
| Liberati et al | 2017 | Advanced Computerized Decision Support Systems (CDSSs)- assist clinicians in their decision-making process, generating recommendations based on up-to-date scientific evidence. | This study explores the barriers and facilitators to the uptake of an evidence-based CDSS as perceived by diverse health professionals in hospitals at different stages of CDSS adoption | Four Hospitals  Italy | - Qualitative - semi-structured interviews | - clinicians’ perceive that the CDSSs may reduce their professional autonomy or may be used against them in the event of medical-legal controversies. - Meanwhile, CDSSs are perceived as a working tool at the service of its users, integrating clinicians’ reasoning and fostering organizational learning. |
| Lin et al | 2018 | redesigned intensive care units (ICUs) | The aim of this study was to explore staff members' perceived effectiveness of a transition from a shared to a single room setting | tertiary teaching hospital  Australia | - Qualitative - Group and individual interviews | - The staff members were part of the team which designed the intervention for this redesign of ICUs - The pre-move ACCESS nurse single room rounding model was helpful for staff to gain insight into what support they may get when working in single rooms, and how to provide support to others. - There was concern that the incidental learning that occurred as nurses learned from each other within a multi-occupancy ICU environment was now lost in a single room environment. |
| Lövestam et al | 2017 | Nutrition Care Process (NCP) and Nutrition Care Process Terminology (NCPT) are currently being implemented by nutrition and dietetics practitioners | The aim of this qualitative study was to explore Swedish dietitians’ experiences of the NCP implementation process in different dietetics environments. | Hospital and primary care  (dietetics workplaces)  Sweden | - Qualitative - seven focus group discussions | - The diversity of dietetics settings and their different prerequisites should be considered in NCP/NCPT implementation strategies. - Different environments pose different challenges which ultimately impact how the NCP is implemented into the clinical care structure. |
| Lowe et al | 2018 | integration of nurse practitioners | The aim of this research was to explore perceptions of organizational change related to the integration of nurse practitioners | Hospitals  Australia | - Qualitative - interviews were undertaken using a purposive sampling strategy of key stakeholders. | - The participants described nurse practitioners as being able to change health care provision by “deliver(ing) the service to the patient” and that “unmet needs are addressed.” - nurse practitioner role are able to coordinate care and improved communication which beneficial to patient care. - There were references to barriers and participants identified funding and budgetary constraints that are hindered by existing practice models. |
| Ludwick et al | 2010 | Electronic medical records | This research aims to explore how remuneration and care setting affect the implementation of electronic medical records (EMRs) | Hospital and primary care  USA | - Qualitative - individually conducted semi-structured interviews | - previous EMR experience affected their decisions about product selection. Other methods of data gathering were product presentations and market scans. - Physicians made their product selections based on a number of factors.   - Products were selected because they supported aggregated patient reporting to be used for identifying patients for recalls.   - Physicians selected systems based on their ability to support interdisciplinary team care.   - Physicians took note of EMRs that supported patient-based task management (ie, a feature that uses the messaging infrastructure of the EMR to delegate patient-related tasks to team members).   - some of our physicians selected products based on the EMR’s ability to support academic research. - Transition to the EMR changed the way the physicians worked. Physicians had to change the way they made encounter notes. They had to learn to fit into the documentation approach dictated by the EMR - Initially, physicians were concerned about patient perception of computer note-taking. Physicians reported that some patients complained that physician attention was focused on the computer. Interestingly, physicians purposefully developed ways of including patients in note-taking. |
| Makowsky et al | 2013 | pharmacists’ adoption of prescribing- model for the Diffusion of Innovations in healthcare services | The aim of this study was to explore factors which influence pharmacists’ adoption of prescribing | Hospitals, Primary care and community  Canada | - Qualitative - Semi-structured telephone interviews to discuss their prescribing practices and explore the facilitators and barriers to implementation | - Pharmacists identified a need for prescribing whether it was switching drugs because of a manufacturer’s shortage, adapting the dose, or stepping in when physicians were not available. - Pharmacists who had adopted prescribing practices had increased their sense of professionalism, the image of the professional healthcare provider and their own job satisfaction and happiness. - Pharmacists were most comfortable prescribing for stable patients on chronic medications who were well known to the pharmacist. - Pharmacists identified the need for enhanced knowledge, skills, and self-efficacy to provide higher levels of patient care including prescribing. - pharmacists who were in patient focused, practice settings were more likely to adopt advanced prescribing practices - Pharmacists stated that physician relationships impacted their prescribing behaviors and individual pharmacists’ decisions to apply for independent prescribing privileges |
| McConnell et al | 2015 | The Liverpool Care Pathway (LCP) for the dying patient | The main aim of this research was to identify the influences that facilitated or hindered successful LCP implementation | health and social care trust  Northern Ireland | - Qualitative - Semi-structured interviews were conducted | - The role of the LCP facilitator was to market the pathway, deliver LCP education and training, and audit how often and well the LCP was being used. - Nursing staff viewed palliative care consultants as a valuable resource for ongoing support and advice, those consultants felt they could not provide adequate support in the context of rapid staff turnover and competing demands - Nurses who worked on wards that were using the LCP believed that the facilitators had successfully embedded the pathway into practice and that the pathway remained an effective part of patient care once the facilitator post had ended. - nurses were active in promoting use of the LCP, while on the medical side palliative care consultants were advocates, medical consultants ambivalent, sometimes skeptical and junior doctors’ attitudes were very much dependent on the approach of their seniors. |
| McGeoch et al | 2015 | Effectiveness of HealthPathways | The aim of this study is to explore perceptions of healthcare professionals on HealthPathways, a website that provides clinical and referral information for general practice teams, relevant to locally available health services and resources | Hospital and primary care  New Zealand | - Online, Quantitative survey - survey questionnaire included questions on the effectiveness and ease-of-use of the website, computer literacy and use of online clinical guidance systems | - The survey questionnaire included questions on the effectiveness and ease-of-use of the website, computer literacy and use of online clinical guidance systems. - Approximately 90–95% of general practice teams considered the website was easy to use and had contributed to both an increase and improvement of care in the community, with about 50% stating that it had improved their relationships with patients and hospital clinicians. - Minor concerns included the website’s increasing size and prescriptive nature and that it increased the duration of a patient consultation. - Approximately 60% of hospital clinicians reported improvements in referral quality and triage and working relationships with general practices since the introduction of HealthPathways. |
| Melnikov et al | 2013 | Converting an open psychiatric ward to a closed one | This study explores the effects of converting an open psychiatric ward to a closed one, (in particular the before–after correlation) among self-efficacy, professional functioning, and uncertainty. | two large psychiatric medical hospitals  Israel | - Quantitative Survey - Two structured pre/postconversion surveys were constructed by the researchers. - respondents were asked to express their feelings and to describe their views with respect to the open-to-closed ward conversion. | - Uncertainty was higher before the conversion than after the conversion. - Professional functioning declined after the conversion. - Self-efficacy was positively correlated with pre- and post-conversion functioning, but negatively correlated with post-conversion uncertainty. |
| Meredith et al | 2015 | Transformation of primary care to new patient-centered models | The aim of this study is to explore Emotional Exhaustion (EE) during the initial phase of national primary care transformation | Primary care  USA | - Online, Quantitative survey - EE subscale of the Maslach Burnout Inventory. Predictors include clinic characteristics (from administrative data) and self-reported efficacy for change, experiences with transformation, and perspectives about the organization | - In total, 53% of PCCs and 43% of staff had high EE. - Primary Care Clinicians (vs. other primary care staff), female (vs. male), and non-Latino (vs. Latino) respondents reported higher EE. - Respondents reporting higher efficacy for change and participatory decision making had lower EE scores. |
| Morrow et al | 2013 | Redesigning postnatal care | This study examines midwives’ views of the changes and their impressions of the effects of the changes on women and their infants. | Hospital  Australia | - Quantitative Surveys - cross-sectional surveys of midwives were conducted six months after the changes to postnatal care were introduced then again, two years later. | - The changes included cessation of routine postnatal observations and the use of clinical pathways for women who gave birth vaginally; promotion of rest through minimal disturbances before 9 am; discouraging the use of the call bell system except in emergency situations; introduction of ‘one-to-one’ time with women; and promotion of normalcy and independence. - Overall, midwives were supportive of, and complied with, the changes to postnatal care. - They agreed that change was needed and believed that the new way of providing care would be better for women and increase individualised care. - Midwives also agreed that the changes would facilitate rest for women, believed that removal of routine observations for women after a vaginal birth was safe and that it would allow more time with women. - Over time, midwives were more likely to feel autonomous when providing postnatal care. - some concerns were raised, mostly in relation to the challenges around postnatal documentation, care provision without the guidance of a care/clinical pathway, and about limiting the use of the call bell to only emergency situations. - Midwives were not confident that the changes would necessarily translate to a measurable increase in women’s satisfaction with care, and were not confident that the changes translated into more time to spend listening and providing support to women. |
| Murray et al | 2012 | Fracture clinic redesign | The aim of this study was to explore the perceptions of emergency room (ER) staff about the impact of the new style clinic on their education, daily practice and interprofessional relations | Hospital  UK | - Quantitative Survey - Survey exploring emergency room (ER) staff perceptions - Adverse events were gathered from the ‘incident record 1’ (IR1) reporting system | - ER staff found the new style clinic was educational, practice changing and improved interprofessional relations, but that it did not interfere with ER duties. - adverse incidents reported fell from 8 per year to 0 per year after the introduction of the new style clinic |
| Musau et al | 2015 | Hospitals have implemented measures related to healthcare-associated infections. | This study examined the effects of healthcare-associated infectious disease outbreaks on nurses’ work | large acute care hospital in Ontario, Canada | - Qualitative - individual interviews | - The incidence rates of methicillin-resistant Staphylococcus aureus (MRSA), Clostridium difficile and vancomycin-resistant enterococci (VRE) at the study site decreased, but remained above provincial benchmarks. - Nurses experienced workload challenges, time pressures and psychological effects stemming from outbreaks and developed various innovations in response. Patient care was also affected. - Participants also acknowledged that certain aspects of their   work, such as documentation, increased during outbreaks, but the nurse–patient ratio did not change in response. Nurses expressed concern that the provision of general nursing care was not completed in a timely manner or at all, particularly when their workloads were very heavy. The nurses cared for isolated patients for long periods. |
| Newman et al | 2016 | Digital Telehealth Network (DTN)  digitisation of a large Australian rural mental health service | The aim of this study was to explore service providers’ experiences of an existing regional telehealth network for mental health care practice twelve months after digitisation in order to identify the benefits of digital telehealth over an analog system for mental health care purposes in rural Australia. | Hospitals  Australia | - Qualitative - interviews and focus groups were conducted | - The main intended use of the DTN was for remote mental health clinical assessments by city-based psychiatrists - Marked differences were reported between sites in the extentof allocating priority DTN use to mental health assessments (rather than other uses); frequency of use varied according to need and the availability of city-based or visiting psychiatrists, from once or twice per day to once a month or less - Overall participants felt that the technical quality of the DTN   was a significant improvement over the previous analog system.   - The DTN has impacted mostly positively on clinical practice,   including improving the speed of practice and the way clinicians communicate   - Some GPs felt that their participation in DTN consultations with psychiatrists exposed them to opportunities to improve clinical practice with mental health interviewing. - Participants observed a range of patient benefits, including improved timeliness/availability of care, reduced travel, improved access to care locally, greater opportunity to receive care without stigma, and reduced family stress. |
| Nilsson et al | 2017 | implementation of value-based healthcare (VBHC) | The aim of this study is to gain a deeper understanding of VBHC when used as a management strategy to improve patients’ health outcomes | University Hospital  Sweden | - Qualitative - qualitative interviews were undertaken | - An example of improvements related to patients’ health outcomes was solving the problem of patients’ nausea. The nurse assistant and the registered nurses started to make investigations to find relevant assessment instruments. Thereafter they started to test how to measure the patients’ nausea. - Improvement related to processes was developing care planning and increasing the number of contact nurses. - Improvement related to measurements was increasing coverage ratio in the National Quality Registers used, and the development of a new coding system for measurements. |
| Nordmark et al | 2016 | Implementation of discharge planning using Normalization Process Theory (NPT) | The aim of this study was to explore the embedding and integration of the Discharge Planning Process (DPP) | Hospital  Sweden | - Multi, written documentation from workshops with staff, registered adverse events and system failure, - web based survey and - individual interviews with staff | - clinicians saw its value in securing the patient’s transition of care from home to the hospital and back home. - They expressed that the quality of the DPP improved with specific discharge planners at the hospital wards. - Registered Nurses (RN) saw the DPP as an extra work task and had to prioritize other work tasks such as patients’ medical treatments, nursing interventions and rounds. RNs expressed that they felt pressure from physicians - They described that information exchange during the DPP depended on the nurses’ individual skills, beliefs and knowledge. A lack of knowledge impeded the DPP. |
| Nyman et al | 2013 | Action Research (AR) to improve hospital based childbirth care, | The aim of this study is to explore midwives' responses to a changed approach in the initial encounters with women and their partners in the labour ward. | Hospital  Sweden | - Qualitative - Individual interviews | - Glancing beyond routines describes how, for some midwives, the changed care approach provided increased potential for them to support each woman and partner, by focusing on their individual needs in a holistic sense. - For some midwives it was better by ‘being confined to own routines ‘capturing a belief that inherent routines were already optimal in the first encounter in the labour ward. |
| Obling | 2013 | accelerated cancer services | This study aims to explore the ways that hospital doctors relate emotions to their understanding of professional medical work and how they respond to recent organisational changes within the field | public teaching hospital Denmark | - Qualitative - semi-structured interviews (n ¼ 14) with doctors from a public teaching hospital | - The doctors represented rich accounts of professional medical work, which includes an understanding of what a doctor should feel and how he/she should make him/herself emotionally available to others. - The impetus for making this appearance was affected by recent new public management reforms and attempts to accelerate the delivery of services. - Some of the interviewees’ identified a certain strategy to manage their feelings that prevent them from becoming emotionally involved with individuals who are serious ill. |
| Pannick et al | 2017 | Prospective clinical team surveillance (PCTS) involves structured interdisciplinary briefings to capture challenges in care delivery, facilitated organizational escalation of the issues they identified, and feedback | The aim of this study is to investigate the impact of prospective clinical team surveillance (PCTS) | Hospitals  UK | - Qualitative - ethnography and two focus groups were conducted with staff taking part in a trial of PCTS. | - Hospital Event Analysis Describing Significant Unanticipated Problems (HEADS-UP)) focused on the problems most commonly identified on medical wards. A single facilitator helped teams advance the issues raised in their briefings, and provided follow-up and feedback to stakeholders throughout the organization. Briefings were known locally as ‘HEADS-UP briefings.’ - the briefings formed a psychologically safe environment in which problems could be discussed openly, without fear of retribution. - The style and timeliness of PCTS feedback also contributed to a sense that this was a non-judgmental forum for team learning - Reflections during the briefing often highlighted the overall management of patient flow, rather than specific actions or diagnostic processes that others could emulate. - PCTS helped identify a route for more rapid resolution of practical problems. It provided an acceptable mechanism for staff to log issues into which they had immediate insight. - PCTS brought about faster resolution of safety and quality issues. There was increased self-monitoring within ward teams. Also, senior ward staff at the daily briefings were more quickly aware of issues they could resolve. |
| Petersen et al | 2018 | e- message system was introduced to ensure dialogue and precise and useful information exchange | The aim of this study is to investigate hospital and home care nurses’ experiences on how an e- message system influences (cross- sectoral) communication | Hospital  Denmark | - Qualitative - Semi- structured focus group interviews and participation observation was conducted | - Nurses used internal systems and journals along with information from patients and relatives to make a comprehensive nursing assessment at admission. Int the se-system, it difficult to understand as the description of the patient’s functional level was rated in a system unfamiliar to them, e.g. practical help was only described in minutes spent on the services. - The home care nurses did not always find the information adequate in the e-message from hospital and often called the hospital for more details - The hospital nurses worked in an environment characterized by heavy workloads, limited access to computers and lack of continuity due to nurses working in shifts. As they did not consider cross- sectoral communication as essential for taking care of patients on the ward, they often found it problematic to prioritize writing reports over taking care of their patient - The e- message system is basically a one- way communication system. When hospital nurses sent information through the e- message system the status of the report changed to “received.” Neither the hospital nurses nor the home care nurses found that the e- message system promoted dialogue between them. |
| Porter et al | 2018 | use of computerised clinical decision support (CCDS) in emergency pre-hospital care | The aim of this study was to explore paramedics’ experience of the CCDS intervention and to identify factors affecting its implementation and us | Ambulance service sites  UK | - Qualitative - interviews and focus groups | - The CCDS was introduced to paramedics during formal training sessions. Experience of adoption and use of the CCDS varied between individual paramedics, with some using it with all eligible patients, some only with patients they thought were ‘suitable’ and some never using it. - Paramedics also encountered problems with printing patient records, finding that not all vehicles were equipped with working printers, paper might be missing, or that having printers fixed to vehicles meant paramedics had to go back to their vehicles to produce print-outs and then return to their patients to give them their copy - connectivity for this web-based system was reported as a problem. - Paramedics reflected on the impact of the CCDS on their practice in relation to patient care and clinical decision-making. Several paramedics discussed how the CCDS contributed to a shift towards a greater role as independent decision-makers, without taking over from their own clinical judgement - Many paramedics held mixed views about the CCDS, reporting benefits but also questioning the extent to which it could assist them with their decision-making |
| Rapson & Kersun | 2014 | Oncology House Physician Model | This study aims to explore the impact of pediatric oncology hospitalist model on the oncology unit staff | Hospital  USA | - Survey - The survey was developed after a literature review of subspecialty hospitalist models - The following domains were explored in the: continuity of care, experience of the hospitalist, efficiency of rounds, handoffs, hospitalist response to nursing, safety, and accessibility of the hospitalist. | - Respondents agreed that house physicians provide better continuity of care - House physicians are more comfortable with the experience level of the physician and are better able to answer questions. - House physicians serve as backup for system-related and patient-related questions and there is an experienced provider was on the floor |
| Rosbergen et al | 2017 | implementation of an enriched environment in an Australian acute stroke unit | This study explores the perceptions and experiences of nursing and allied health professionals involved in the implementation of an enriched environment in an Australian acute stroke unit | Hospital  Australia | - Qualitative - Face-to-face, semi-structured interviews | - The staff perceived that ‘the road to recovery had started’ for patients. An enriched environment was described to shift the focus to recovery in the acute setting, which was experienced through increased patient activity, greater psychological well-being and empowering patients and families - It was described that it takes a team’ to successfully create an enriched environment. Integral to building the team were positive interdisciplinary team dynamics and education. The impact of the enriched environment on workload was diversely experienced by staff. - Staff reflected that changing work routines was difficult. Contextual factors such as a supportive physical environment and variety in individual enrichment opportunities were indicated to enhance implementation. - Education was perceived of great importance to successfully implement an enriched environment. Staff expressed that the interactive educational workshops that were provided prior to embedding the enriched environment created a basic understanding of the concept and awareness of the different components of an enriched environment. |
| Russ et al | 2010 | implemented electronic health record (EHR) | The aim of this study was to explore helpful and challenging aspects of electronic health information with respect to clinical workflow and identify a set of characteristics that support patient care processes. | Hospital  USA | - Qualitative - Interviews | - Participants provided examples of how electronic information was not consistent within Health information technology (HIT), between HIT and paper documents, and also between HIT and individuals’ personal knowledge. - Obtaining current electronic information was sometimes challenging - A nurse practitioners (NP) described how electronic information was not always complete - In some cases, incorrect information hindered clinical workflow. Examples involved incorrect note titles, inaccurate medication lists, and problems with patient phone numbers. - Participants discussed automatic electronic logouts; referral forms sent to outside clinics via the virtual private network (VPN); and future technologies for electronic signatures - Interviews revealed accessibility problems when secure login processes were too long, systems were running slow, or computers were down. Accessibility issues were reported by clinical workers and support staff. |
| Samaranayake et al | 2014 | Implementing a bar-code assisted medication administration (BCMA) system | The aim of this study is to explore the effects of a bar-code assisted medication administration system used without the support of computerised prescribing (stand-alone BCMA), on the dispensing process and its users. | Hospital  Hong Kong | - Qualitative interviews and observation | - Most pharmacy staff believed that the dispensing process was slower after implementing the BCMA system - Some participants thought that work was made more difficult or complicated after implementing the technology than before - Some participants viewed that the absence of computerized prescribing was a barrier for prompt updating of patient profiles when prescribing changes were made - the pharmacy staff believed that the new system   improved the safety in the drug administration process and benefited the nursing staff and patients |
| Solhaug et al | 2010 | The Newborn Individualized Developmental Care and Assessment Program (NIDCAP) | The aim of this study was to explore the staff perceptions of implementation of The Newborn Individualized Developmental Care and Assessment Program (NIDCAP) | University Hospital  Norway | - Survey and Qualitative - This survey instrument was developed by Swedish experts and has been used to survey staff opinion after NIDCAP implementation in several European countries such as Sweden, France and The Netherlands - The questions were related to perceptions of infant well-being, parental participation and staff working conditions | - staff considered NIDCAP to have a positive impact on infant well being as well as on their opportunities to rest and sleep during the hospital stay - staff considered NIDCAP to have a positive impact on infant well being as well as on their opportunities to rest and sleep during the hospital stay - Staff in particular perceived that NIDCAP had a positive impact on their capability to influence infant well-being. - The nurses were of the view that the structure and language in the care plans was seen as very simple. it was agreed that the observation reports to a large extent contributed to continuity of care, and that the care recommendations were helpful. |
| Sommerbakk et al | 2016 | IMPACT project (IMplementation of quality indicators in PAlliative Care sTudy) | This study aims to identify factors perceived as barriers or facilitators for improving Palliative Care )PC) in cancer and dementia | two hospitals, one nursing home, and two local primary care  Norway | - Qualitative - Individual, dual-participant and focus group interviews | - One challenge to implementing PC tools was that few tools are specifically tailored to primary care. - The use of information collected in the forms motivated staff to use them - Several participants said that having the new tools integrated into the electronic system would then be easily accessible to staff. - Lack of knowledge about and professional skills in PC were mentioned by several interviewees as barriers to improving PC in the services. - Several nurses expressed that they felt anxious about being responsible for terminally ill patients. Training in PC was reported as an important measure to improve the confidence of staff - A barrier to a constructive culture of change was lack of support from colleagues. Staff members did not understand why some of their colleagues were given more training and responsibilities that took them away from daily clinical work. This lack of understanding from colleagues had been stressful for staff members involved in improvement processes. - staff had more administrative work to do in addition to the clinical work. The clinical workload had also increased because the patients admitted to all the services were generally in poorer condition than before. |
| Southgate et al | 2011 | Workplace nurses’ injury and return-to-work | The purpose of this study was to identify the factors that facilitate or impede the successful return to work (RTW) of nurses who have sustained a workplace injury | Hospital  Australia | - Qualitative - focus group discussions | - scarcity of qualified nurses had led to some healthcare employers treating nurses as a valuable resource, even when they were injured-workplace were keen to retain injured nurses due to workforce shortages. - It was emphasized to provide nurses with suitable duties and to incorporate clinical or patient-centered duties within RTW plans. They also emphasized that RTW plans need to meet individual life circumstances. Knowledge of the nurse’s life circumstances is acquired by forging an early supportive relationship with the injured nurse. |
| Stahl et al | 2017 | Picker Employee Questionnaire | The aim of this research is to validate and adapt the Picker Employee Questionnaire With Hospital Midwives | Hospital  Germany | - Quantitative- Survey - Study on adaptation and validation of the questionnaire | - The Picker Employee Questionnaire was originally designed as a general measure for use by all hospital staff. - The adaption of the Picker Employee Questionnaire resulted in a tool with 75 closed questions referring to central aspects of work environment, experience, and engagement. - The questionnaire was suited for the measurement of midwives’ work experience, environment, and engagement. - It is a useful tool that supports in shaping and motivating an efficient work environment for midwives. |
| Van Doormaal et al | 2010 | Computerized physician order entry (CPOE) system | The aim of this study is to explore physicians’ and nurses’ expectations before and experiences after the implementation of a computerized physician order entry (CPOE) system | Hospitals  Netherlands | - Quantitative- Survey - expectations and experiences of physicians and nurses with the CPOE system were measured with statements on a 5-point Likert scale (1 = completely disagree, 5 = completely agree). | - Two semi-structured questionnaires were developed targeting physicians and nurses respectively. These surveys were constructed to measure expectations and to measure experiences with CPOE. - Physicians had positive expectations of CPOE being able to reduce prescribing errors and to give an improved overview of patients’ medication use which was in line with their experience with CPOE - Nurses experienced CPOE to improve the clarity of the prescriptions just as they had expected |
| Wall | 2014 | Self-employed nurses as change agents in healthcare | This research was an ethnographic research that investigated how self-employed nurses perceive the contemporary healthcare field, what attributes they possess that facilitate their roles as change agents, what strategies they use to influence change, and what consequences they face for their actions. | Hospitals and private practice  Canada | - Mixed, focused ethnography/observation - Qualitative - Self-employed nurses were sought for their point of view as possible change agents uniquely positioned within an institutionalized field | - the nurses in this study perceived overwhelming and disheartening issues in the healthcare system- they had concerns about institutionalized ideas about economics, efficiency, and reactive illness care, which contributed to a lack of real reform in the system. - A concern was raised about budget consciousness by the hospital which thus eliminated some important patient care services - role in community health education was eliminated because health promotion and education is easily dropped off the slate in favor of hospital services - Self-employed nurses had a special level of character development and individuation and a sense of awakening which was distinct from nurses in general. They were more clinically involved and ready to take risks. The nurses viewed themselves as risk takers, open to change and positioned to promote innovation in healthcare delivery. - Self-employed nurses expand the professional jurisdiction of nursing, and called upon their existing professional knowledge and standards to adapt nursing - Some started their own practice centers and began to broaden their professional potential. - these nurses began to exercise a vision of healthcare that went beyond hospital-centered care. |
| Weber et al | 2011 | In England emergency departments (EDs) must either discharge patients or place them in a hospital bed within 4 hours of arrival | This study aimed to explore the within four hours model of care | Hospital  UK | - Qualitative - Semi-structured interviews | - Respondents believed the target offered an opportunity to improve care for patients - An important concern was that the wards had not changed their processes sufficiently to be able to quickly institute continuing evaluation and treatment on patients arriving from the ED - Meeting the 4-hour target challenged departments to accept sweeping changes in traditional ways of working. - The 4-hour target was a challenge for ED nurses. In addition to caring for patients within the time allotted, nurses kept an eye on the clock, prodded physicians for decisions, and reported delays to senior management or more senior clinicians. There was often a conflict between ED and ward nursing staff. - The ED nurses were empowered to initiate diagnostic testing when patients arrived in the ED and to contact more senior specialists when there were delays in obtaining consultations. |
| White et al | 2014 | Intensive Care Unit Patients in the Post-anesthesia Care Unit | The purpose of this study was to understand the experiences of post-anesthesia nurses caring for intensive care unit (ICU) patients in the post-anesthesia care unit (PACU). | Hospital  Canada | - Qualitative - Case study | - Participants were functioning as both a PACU nurse and an ICU nurse at the same time - Participants were functioning as both a PACU nurse and an ICU nurse at the same time-participants felt as if they were giving less than the best care- they felt that they should be doing but felt incapable of doing it. - Nurses felt a gap in their knowledge about high-quality care which is provided in the ICU |
| Ziebert et al | 2016 | Transition into Practice | This study explored the experiences of all newly hired nurses (at 3, 6, and 12 months post-hire) during a newly designed transition-to-practice program at a pediatric hospital. | Hospital  USA | - Qualitative - Interviews | - Participants appreciated opportunities for feedback and reciprocal communication - Unit socialization was important, and participants could identify the value of feeling welcomed and recognized. - They also felt they were well-supported in their new roles by seniors checking with them on their progress. |
