# Supplementary material for: Clinicians’ experience of providing care: a rapid review
Source: BMC Health Serv Res. 2020 Oct 15;20:952. doi: 10.1186/s12913-020-05812-3 (PMC7559170; doi:10.1186/s12913-020-05812-3)
Supplement: Supplementary file 3 — Additional file 3. [file 12913_2020_5812_MOESM3_ESM.docx]

**Appendix 3: Electronic search strategy**

**Database(s): Ovid MEDLINE(R) ALL 2010 to June 26, 2019 
Search Strategy**:

|  |  |
| --- | --- |

Search Strategy:

| **#** | **Searches** | **Results** |
| --- | --- | --- |
| 1 | exp hospitals/ or exp hospital departments/ or Emergency Service, Hospital/ | 411649 |
| 2 | (acute care hospital* or acute hospital*).mp. or Hospital?.ti,ab. or Emergency Department*.mp. [mp=title, abstract, original title, name of substance word, subject heading word, floating sub-heading word, keyword heading word, organism supplementary concept word, protocol supplementary concept word, rare disease supplementary concept word, unique identifier, synonyms] | 1097356 |
| 3 | Medical Staff, Hospital/ or Nursing staff, hospital/ or Dental Staff, Hospital.mp. or Hospital staff.mp. [mp=title, abstract, original title, name of substance word, subject heading word, floating sub-heading word, keyword heading word, organism supplementary concept word, protocol supplementary concept word, rare disease supplementary concept word, unique identifier, synonyms] | 68130 |
| 4 | 1 or 2 | 1284646 |
| 5 | exp health personnel/ or exp Patient Care Team/ | 539427 |
| 6 | (Clinical staff or Clinicians or Health professionals or Allied health or Medical staff or Physicians or Midwife* or Midwive* or Nurses or Nursing staff or Health care professional*).mp. or Nursing.hw. [mp=title, abstract, original title, name of substance word, subject heading word, floating sub-heading word, keyword heading word, organism supplementary concept word, protocol supplementary concept word, rare disease supplementary concept word, unique identifier, synonyms] | 1148864 |
| 7 | 5 or 6 | 1357635 |
| 8 | Diffusion of innovation/ or Health Plan Implementation/ or Hospital Restructuring/ or Organizational Innovation/ or Personnel Downsizing/ | 48723 |
| 9 | (change management or downsize* or organi?ational change* or organi?ational reform* or organi?ational restructure* or practice change or structural change*).mp. [mp=title, abstract, original title, name of substance word, subject heading word, floating sub-heading word, keyword heading word, organism supplementary concept word, protocol supplementary concept word, rare disease supplementary concept word, unique identifier, synonyms] | 46931 |
| 10 | 8 or 9 | 94060 |
| 11 | Attitude of health personnel/ or Experience*.mp. or Perspective*.mp. or Perception*.mp. or Reaction*.mp. or Perceived.mp. or Focus group*.mp. or Change fatigue.mp. [mp=title, abstract, original title, name of substance word, subject heading word, floating sub-heading word, keyword heading word, organism supplementary concept word, protocol supplementary concept word, rare disease supplementary concept word, unique identifier, synonyms] | 3373064 |
| 12 | 3 and 10 and 11 | 1013 |
| 13 | 4 and 7 and 10 and 11 | 1895 |
| 14 | 12 or 13 | 2286 |
| 15 | limit 14 to (abstracts and english language) | 1999 |
| 16 | limit 15 to yr="2010 -Current" | 740 |
|  | To include developed countries of interest |  |
| 17 | (developed countries or european union).af. | 78235 |
| 18 | europe/ or andorra/ or austria/ or belgium/ or exp france/ or exp germany/ or exp united kingdom/ or greece/ or ireland/ or exp italy/ or liechtenstein/ or luxembourg/ or monaco/ or netherlands/ or portugal/ or exp "scandinavian and nordic countries"/ or spain/ or switzerland/ or exp australia/ or new zealand/ | 1302040 |
| 19 | exp canada/ | 150370 |
| 20 | (united kingdom or england or scotland or wales or denmark or finland or iceland or norway or sweden).af. | 7752198 |
| 21 | (canada or oecd).af. | 1014168 |
| 22 | (europe or andorra or austria or belgium or france or germany or greece or ireland or italy or liechtenstein or luxembourg or monaco or netherlands or portugal or spain or switzerland or australia or new zealand).af. | 10027348 |
| 23 | or/17-22 | 16583900 |
| 24 | 16 and 23 | 517 |
| 25 | remove duplicates from 34 | 663 |
